# Flavonoid glycosides isolated from *Epimedium brevicornum* and their estrogen biosynthesis-promoting effects

**DOI:** 10.1038/s41598-017-08203-7

**Published:** 2017-08-10

**Authors:** Fu Li, Bao-Wen Du, Dan-Feng Lu, Wen-Xuan Wu, Kanjana Wongkrajang, Lun Wang, Wen-Chen Pu, Chang-Lu Liu, Han-Wei Liu, Ming-Kui Wang, Fei Wang

**Affiliations:** 1 Chengdu Institute of Biology, Chinese Academy of Sciences, Chengdu, 610041 P. R. China; 2 Key Laboratory of Mountain Ecological Restoration and Bioresource Utilization and Ecological Restoration Biodiversity Conservation, Key Laboratory of Sichuan Province, Chengdu Institute of Biology, Chinese Academy of Sciences, Chengdu, 610041 P. R. China; 3 Key Laboratory of Animal Models and Human Disease Mechanisms of Chinese Academy of Sciences & Yunnan Province, Kunming Institute of Zoology, Chinese Academy of Sciences, Kunming, 650223 P. R. China; 4 Department of Chemistry, Faculty of Science and Technology, Pibulsongkram Rajabhat University, Phitsanulok, 65000 Thailand; 5 Key Laboratory of Exploitation and Study of Distinctive Plants in Education Department of Sichuan Province, Sichuan University of Arts and Science, Dazhou, 635000 P. R. China; 6 Ningbo Entry-Exit Inspection and Quarantine Bureau Technical Center, Ningbo, 315012 P. R. China; 7 University of Chinese Academy of Sciences, Beijing, 100049 P. R. China

## Abstract

*Epimedium brevicornum* Maxim has a long history of use in the treatment of estrogen deficiency-related diseases. However, the chemical constituents and mechanism of action of this medicinal plant are not fully understood. In the present study, we isolated four new isoprenylated flavonoid glycosides, as well as 16 known flavonoids (13 isoprenylated flavonoids), from this plant. The chemical structures of the new flavonoid glycosides were elucidated by extensive spectroscopic analysis. The new compounds **1**–**4** were potent promoters of estrogen biosynthesis in human ovarian granulosa-like KGN cells. ZW1, an isoprenylated flavonoid analogue and a specific inhibitor of phosphodiesterase 5 (PDE5), was synthesized and used to explore the mechanism of the isoprenylated analogues on estrogen biosynthesis. ZW1 treatment increased estrogen production by upregulation of aromatase mRNA and protein expression. ZW1 increased the phosphorylation of cAMP response element-binding protein (CREB). Further study showed that the inhibition of PDE5 by ZW1 increased estrogen biosynthesis partly through suppression of phosphodiesterase 3 (PDE3). Our results suggested that the isoprenylated flavonoids from *E. brevicornum* may produce beneficial health effects through the promotion of estrogen biosynthesis. PDE5 warrants further investigation as a new therapeutic target for estrogen biosynthesis in the prevention and treatment of estrogen-deficiency related diseases.

## Introduction

Estrogens play a crucial role in the normal physiology of a variety of tissues, including the mammary glands, reproductive tract, central nervous system, and skeleton. The homeostasis of estrogen in humans requires fine-tuning to maintain appropriate levels in tissues to allow execution of its intended functions; disturbance of this balance can lead to the occurrence of a variety of diseases^1^. For example, overexposure to estrogen results in breast, ovarian, and endometrial carcinogenesis, whereas estrogen deficiency can lead to amenorrhea, infertility, osteoporosis, atherosclerosis, and Alzheimer’s diseases. In humans, the biosynthesis of estrogen is catalyzed by aromatase, which converts androgens to estrogens in various tissues including ovary, bone, testis, placenta, brain, and adipose tissue. Although the coding region of an aromatase remains unchanged, the transcriptional control of aromatase is tissue-specific because of the presence of different promoters under the control of different hormones, cytokines, and other factors^2^. Although estrogen supplementation is an established regimen for the treatment of estrogen deficiency, its clinical use is limited by the side effects associated with long-term use, such as increased risk of breast, ovarian, and endometrial cancers^3^. Thus, alternative methods that can improve the therapeutic efficacy and safety with local promotion of estrogen biosynthesis should be developed for the prevention and treatment of diseases caused by estrogen deficiency.

The major site of estrogen biosynthesis in premenopausal women is the ovary, where aromatase expression results from the activation of its proximal promoter (promoterI.3/II)^4^. The primary signaling cascade through which this promoter is regulated is the cAMP/protein kinase A (PKA)/cAMP response element-binding protein (CREB) pathway^5^. CREB is the principal regulatory component in the regulation of the aromatase gene, which is stimulated by follicle-stimulating hormone (FSH)-activated cAMP-dependent PKA. Recently, it has been shown that the cyclic 3′,5′-guanosine monophosphate (cGMP)-dependent signaling pathway also mediates a wide range of influences on the ovary^6^. The cGMP is mainly hydrolyzed by phosphodiesterase 5 (PDE5), dysregulation of which is involved in occurrence of a variety of diseases including male and female sexual dysfunction^7^. PDE5 inhibition by tadalafil and sildenafil, two specific inhibitors of PDE5 used for the treatment of erectile dysfunction, promoted estrogen production by an increase of aromatase expression through activation of the cGMP/PKG pathway in human adipocytes^8^. Moreover, sildenafil was found to significantly increase cAMP and suppress the spontaneous maturation of mouse oocytes, suggesting there is cross-talk between cGMP and cAMP signals in this process^9^. The role and mechanism of PDE5 in the mediation of estrogen biosynthesis in the ovaries is still unclear, and uncovering this may be helpful for the development of new therapeutic means for the modulation of estrogen production in the ovaries.

Many medicinal plants have been shown to regulate ovulation and improve the uterine blood flow and menstrual changes of the endometrium^10^. Flavonoids, one of the major components in these plants, are considered to exert beneficial effects on human health by modulation of the estrogen receptor^11^. Flavoinds are also considered to be a good source of aromatase inhibitors with low associated toxicity, however, their effects on the transcriptional control of aromatase in ovary are rarely studied^12, 13^. *Epimedium brevicornum* Maxim is a medicinal plant that has been used in various traditional Chinese formulations for thousands of years. It is also a component of various modern proprietary traditional Chinese medicine products for the treatment of cardiovascular disease, infertility, impotence, amnesia, lumbago, arthritis, and numbness and weakness of the limbs^14^. *E. brevicornum* and its major bioactive constituent icariin have been reported to presents various pharmacological actions including osteoprotection, neuroprotection, cardiovascular protection, anti-inflammation, immunoprotection, and reproductive function^15^. In a previous study, we found the extract of *E. brevicornum* and icariin promoted the production of estrogen in human ovarian granulosa cells and osteoblastic cells by upregulation of aromatase expression, providing new insight into the mechanism behind their pharmacological effects^16^. However, it is still unclear whether other compounds extracted from this medicinal plant have similar effects on estrogen biosynthesis and what the mechanism of action of these compounds would be. In the present study, we isolated compounds from *E. brevicornum* and examined the effect and underlying mechanism of estrogen biosynthesis-promoting compounds on aromatase expression.

## Results

### Structural elucidation of compounds 1–4

**Figure 1:**
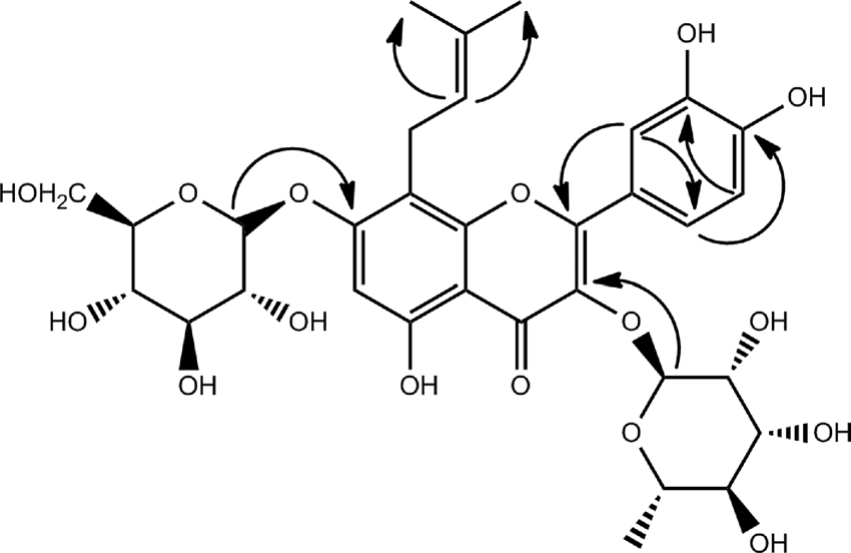
Key HMBC correlations of compound **1**.

**Figure 2:**
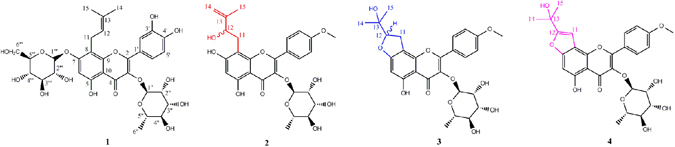
Chemical structures of new compounds **1–4**.

3′-Hydroxyl epimedoside A (**1**), a yellow powder with a molecular formula of C_32_H_38_O_16_ deduced from HRESIMS (*m/z* 677.2095, calcd for [M − H]^−^ 677.2087), required 14 degrees of unsaturation. The ^1^H-NMR data (Table S1) suggested the existence of characteristic isopentene group signals at δ_H_ 1.58 (3 H, s), 1.67 (3 H, s), 5.13 (1 H, t, *J* = 4.8 Hz), 3.51 (1 H, m, overlapped), and 3.59 (1 H, m, overlapped). The presence of two sugar residues was deduced from the appearance of anomeric hydrogen signals at δ_H_ 4.95 (1 H, d, *J* = 6.0 Hz) and 5.23 (1 H, d, *J* = 1.2 Hz). The characteristic carbon signals (Table S1) at δ_C_ 18.3 and 61.1, together with the hydrogen signals at δ_H_ 0.80 (3 H, d, *J* = 6.4 Hz) and the two anomeric hydrogen signals, indicated that these two sugar residues were methylpentose and hexose, which were further confirmed as L-rhamnose and D-glucose, respectively, using a previously reported method^17^. The coupling constant of the anomeric hydrogen suggested a *β* configuration for the glucose moiety, while the configuration for the rhamnose unit was determined by the chemical shifts of C-3′′ and C-5′′^18^. The UV absorption bands at 259 nm and 354 nm, along with NMR data, demonstrated that compound **1** possessed a flavonoid skeleton. The NMR data of compound **1** were similar to those of epimedoside A^19^, except that a 1,3,4-trisubstituted phenyl group, based on an ABX spin coupling system at δ_H_ 6.83 (1 H, d, *J* = 8.4 Hz), 7.32 (1 H, d, *J* = 1.6 Hz), and 7.27 (1 H, dd, *J* = 8.4, 1.6 Hz) in compound **1**, replaced the 1,4-disubstituted phenyl group, based on an A_2_B_2_ spin coupling system in epimedoside A, indicating the presence of a hydroxyl group at C-3′ in compound **1**. HMBC correlations (Fig. 1) of H-1′ (δ_H_ 5.23) and H-1′′ (δ_H_ 4.95) with the quaternary carbons at δ_C_ 134.6 (C-3) and δ_C_ 160.8 (C-7), respectively, further confirmed the linkage of the rhamnose residue to C-3 and glucose residue to C-7. Accordingly, the structure of compound **1** was identified as shown (Fig. 2).

Neo-sagittasine A (**2**), a yellow powder, had a pseudo-molecular ion at *m/z* 529.1720 (calcd for [M − H]^−^ 529.1715), suggesting a molecular formula of C_27_H_30_O_11_. The UV absorption bands at 269 nm and 320 nm suggested compound **2** was a flavonoid glycoside. The ^1^H-NMR data (Table S2) showed A_2_B_2_-type signals at δ_H_ 7.08 (2 H, d, *J* = 8.6 Hz) and 7.92 (2 H, d, *J* = 8.6 Hz), an aromatic proton singlet at δ_H_ 6.28 (s), and a methoxy group at δ_H_ 3.83 (3 H, s). The existence of a rhamnose residue was deduced from the typical signals at δ_H_ 5.32 (1 H, d, *J* = 1.2 Hz) and 0.71 (3 H, s), respectively. In addition, the appearance of ^1^H-NMR signals due to one methyl group, one methylene group, two terminal double bond protons, and an oxygen-bearing methine proton implied the presence of an isopentene group. The ^1^H-NMR and^13^C-NMR spectra of **2** (Table S2) resembled those of sagittasine A^20^, except for the presence of a sugar moiety in place of the three sugar units in sagittasine A. The HMBC correlations further confirmed that compound **2** and sagittasine A shared an identical aglycone. Unfortunately, the data were not sufficiently conclusive to determine the absolute configuration of C-12. The HMBC correlation between the anomeric proton (δ_H_ 5.32) and C-3 (δ_C_ 134.5) supported the attachment of the rhamnose residue to C-3 of the aglycone. Therefore, the structure of compound **2** was elucidated as shown (Fig. 2).

Dihydrofuran-baohuoside I (**3**), a yellow powder, gave a pseudo-molecular ion at *m/z* 529.1724 (calcd for [M − H]^−^ 529.1715), indicating a molecular formula of C_27_H_30_O_11_. The ^1^H-NMR and ^13^C-NMR data of **3** (Table S3) were similar to **2**, except for the signals due to the side chain at C-8. The appearance of two methyl groups at δ_H_ 1.13 (3 H, s) and 1.14 (3 H, s) in the ^1^H-NMR spectrum, along with a quaternary carbon at δ_C_ 70.5 in **3**, instead of those attributed to a double bond in **2** in the ^13^C-NMR spectrum, demonstrated the presence of a (CH_3_)_2_-C(OH)- unit. The downfield chemical shift of C-12 from δ_C_ 73.9 in **2** to δ_C_ 92.1 in **3**, together with the need for 13 degrees of unsaturation, suggested the cyclization between C-12 and C-7 through an oxygen atom. The occurrence of the furan ring was further confirmed by the HMBC correlation of H-12 (δ_H_ 4.77) with C-7 (δ_C_ 166.8). Additionally, the absolute stereochemistry of C-12 in **3** could not be determined using NMR data. Based on the above evidence, the structure of compound **3** was determined as shown in Fig. 2.

The molecular composition of furan-baohuoside I (**4**), C_27_H_28_O_11_, was deduced from HRESIMS (*m/z* 551.1516, calcd for [M + Na]^+^ 551.1524), and required 14 degrees of unsaturation. The ^1^H-NMR data of **4** (Table S4) were similar to those of **3** except for an olefinic proton at δ_H_ 7.00 (1 H, s) in **4** instead of a methyne proton at δ_H_ 4.77 and two protons at δ_H_ 3.16 and 3.26 due to a methylene group in **3**, indicating the presence of a double bond between C-11 and C-12. The HMBC correlation of H-11 with C-9 further confirmed the location of the double bond. Thus, the structure of compound **4** was identified as shown in Fig. 2.

The known compounds obtained in this experiment were identified as icariin (**5**)^21^, epimedin A_1_ (**6**)^22^, epimedin A (**7**)^22^, epimedin B (**8**)^22^, epimedin C (**9**)^22^, epimedoside A (**10**)^23^, diphylloside C (**11**)^24^, epimedoside E (**12**)^25^, icariside II (**13**)^21^, diphylloside B (**14**)^25^, icariside I (**15**)^26^, luteolin (**16**)^27^, icaritin-3-O-α-L-rhamnoside (**17**)^28^, kaempferol-3-O-α-L-rhamnoside (**18**)^29^, caohuoside C (**19**)^30^, and kaempferol-3-O-(2-β-D-glucopyranosyl)-α-L-rhamnopyranoside-7-O-α-L-rhamnopyranoside (**20**)^31^ (Fig. S1) by comparison of the NMR and MS spectra with those reported in the literature.

### Effects of natural products from *E. brevicornum* on estrogen biosynthesis

**Figure 3:**
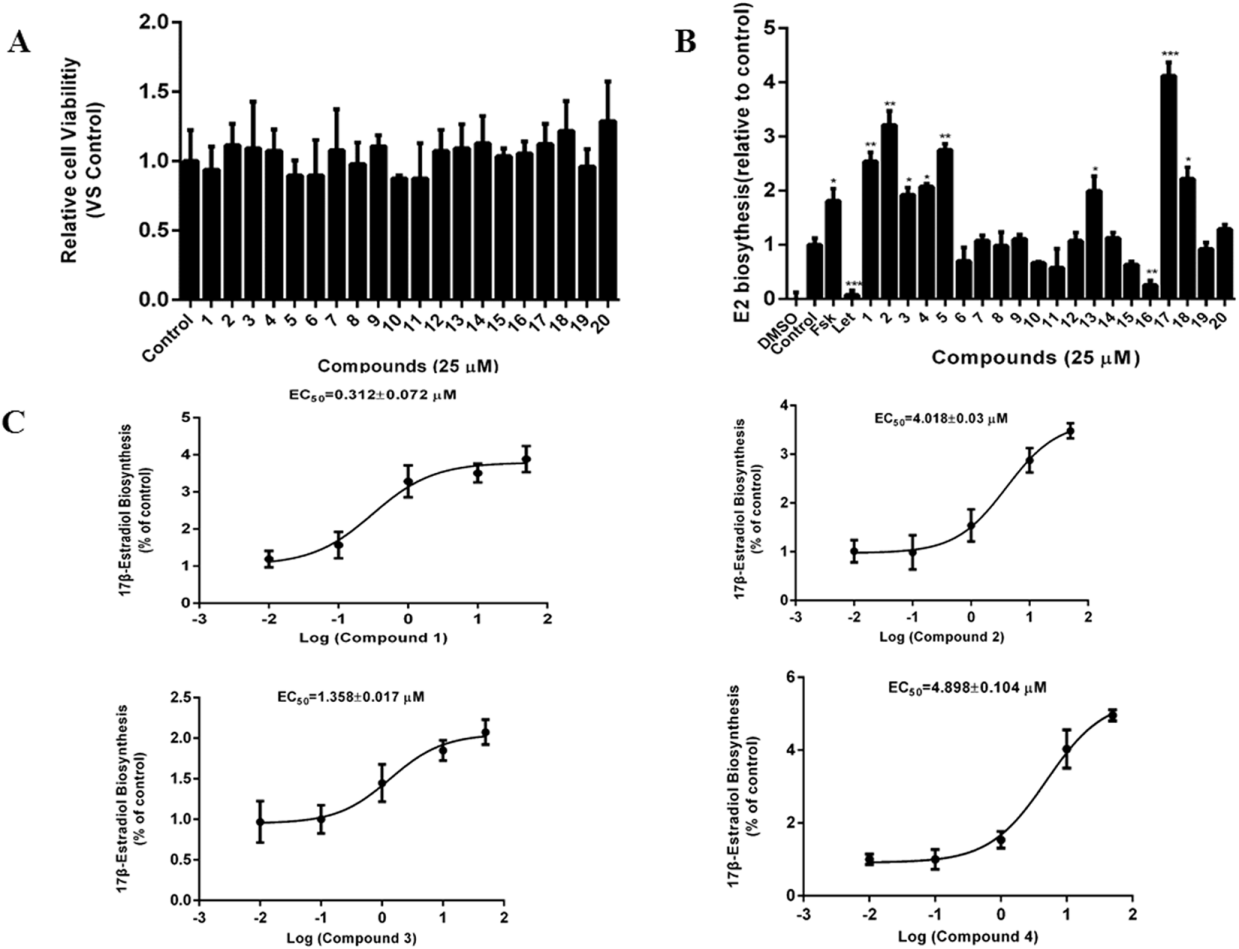
Effects of the isolated compounds on estrogen biosynthesis. (**A**) KGN cells seeded in 96-well plates were pretreated with the test compounds at 10 μg/mL for 24 h. Alamar Blue reagent was added and the plates were incubated for a further 4 h, after which the fluorescence intensities were measured. (**B**) KGN cells seeded in 24-well plates overnight were pretreated with the test compounds for 24 h. Subsequently, the cells were supplemented with 10 nM testosterone for a further 24 h. The concentration of 17β-estradiol in the culture medium was quantified using a 17β-estradiol magnetic particle-based ELISA. (**C**) The concentration-response curve of compounds **1**–**4** for promotion of estrogen biosynthesis in KGN cells. FSK, 10 μM forskolin; Let, 1 μM letrozol. *p < 0.05, **p < 0.01, ***p < 0.001 compared with the control (*n* = 3).

To test the cytotoxicity of isolated compounds against human granulosa-like KGN cells, we pretreated these cells with each of the compounds for 24 h, and then analyzed the cells with an Alamar Blue assay. As shown in Fig. 3A, the compounds were not cytotoxic to KGN cells. Next, the effects of all the isolated compounds on estrogen biosynthesis in KGN cells were examined. As shown in Fig. 3B, forskolin (FSK), an adenylate cyclase agonist that activates the PKA/CREB pathway, significantly promoted 17β-estradiol production, whereas letrozole (Let), an aromatase inhibitor, significantly inhibited 17β-estradiol production. Among the 20 compounds, eight showed promotive effects on the biosynthesis of estrogen, including 3′-hydroxyl epimedoside A (**1**), neo-sagittasine A (**2**), dihydrofuran-baohuoside I (**3**), furan-baohuoside I (**4**), icariin (**5**), icariside II (**13**), icaritin-3-O-α-L-rhamnoside (**17**), and kaempferol-3-O-α-L-rhamnoside (**18**). Consistent with our previous report^32^, luteolin (**16**) inhibited the biosynthesis of estrogen. In addition, the four new compounds (**1–4**) increased estrogen production in a concentration-dependent manner (Fig. 3C), with calculated EC_50_ values of 0.312 μM, 4.018 μM, 1.358 μM, and 4.898 μM respectively.

### Icariin derivative ZW1 promotes estrogen biosynthesis in KGN cells

**Figure 4:**
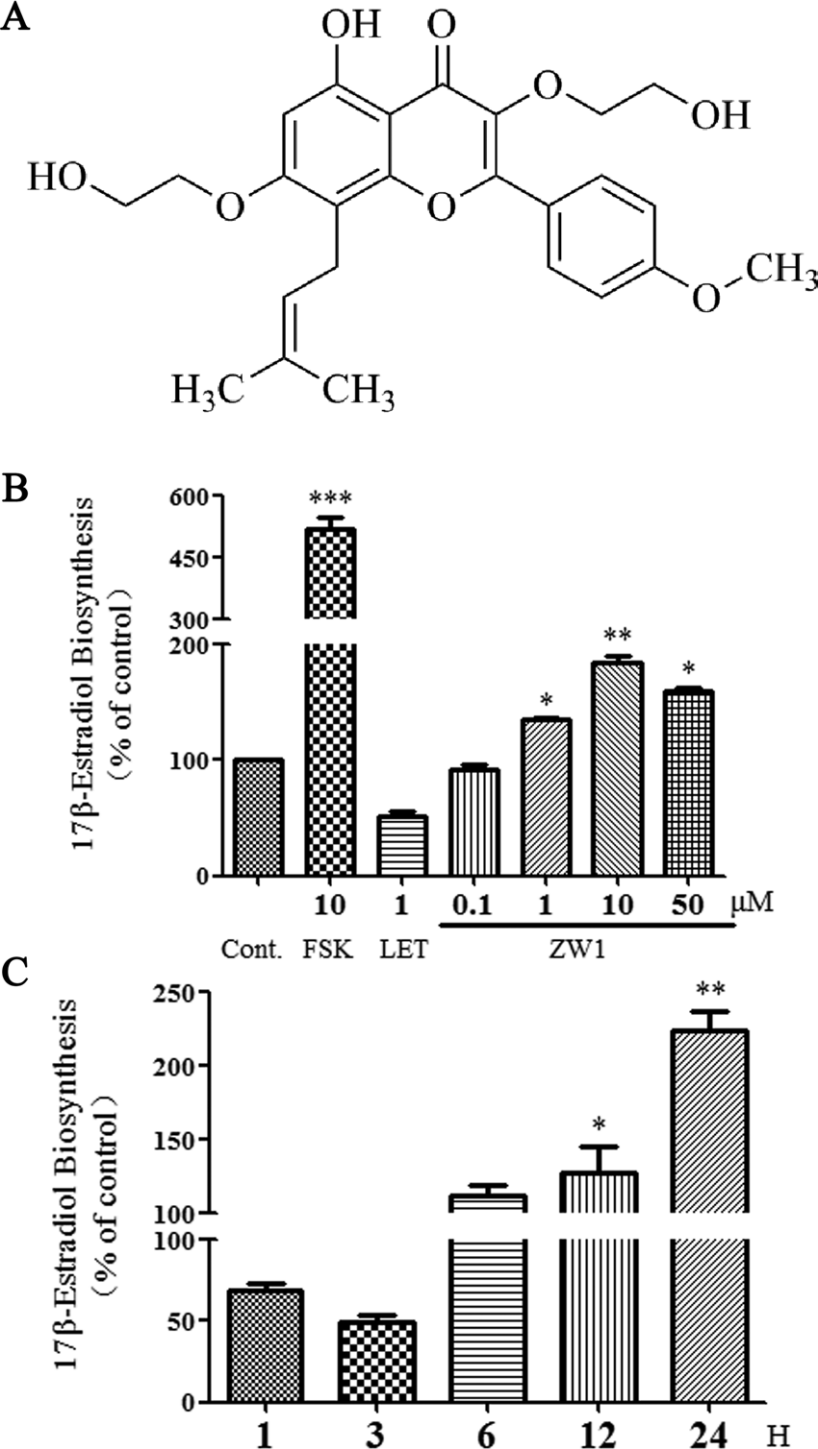
The effect of ZW1 on estrogen biosynthesis. (**A**) The chemical structure of icariin derivative ZW1. (**B**) KGN cells seeded in 24-well plates and left overnight were pretreated with the test compounds for 24 h. Subsequently, the cells were supplemented with 10 nM testosterone for a further 24 h. The concentration of 17β-estradiol in the culture medium was quantified using a 17β-estradiol magnetic particle-based ELISA. (**C**) The time course of the induction of estrogen biosynthesis in KGN cells by 10 μM ZW1. Cont., DMSO-treated control; FSK, 10 μM forskolin; LET, 1 μM letrozol. *p < 0.05, **p < 0.01 compared with the control (*n* = 3).

Further elucidation of the mechanism of these bioactive compounds on estrogen biosynthesis was limited by the available amount of compounds. In our previous study, we found that sildenafil, a specific inhibitor of PDE5, promotes estrogen biosynthesis in human granulosa-like KGN cells^14^. Icariin, icariside II, and their natural analogues have also been identified as PDE5 inhibitors^33, 34^. To demonstrate whether icariin and its analogues promote estrogen biosynthesis through PDE5 inhibition, we synthesized an icariin derivative, 3,7-bis(2-hydroxyethyl)icaritin (ZW1), which has been demonstrated as a more potent PDE5 inhibitor than icariin. The chemical structure of ZW1 is shown in Fig. 4A. KGN cells were pretreated for 24 h with various concentrations of ZW1 followed by incubation with testosterone for 24 h. As shown in Fig. 4B, ZW1 concentrations of 1–50 μM significantly increased the production of 17β-estradiol in a concentration-dependent manner in KGN cells compared with the DMSO-treated control. The promotion of 17β-estradiol biosynthesis by ZW1 at a concentration of 10 μM was first evident at 12 h and was most evident at 24 h when 17β-estradiol biosynthesis was enhanced approximately 2.2-fold compared with the control (Fig. 4C). These results indicate that the icariin derivative ZW1 could potently promote the biosynthesis of estrogen in human ovarian granulosa cells.

### ZW1 increases aromatase expression in KGN cells

**Figure 5:**
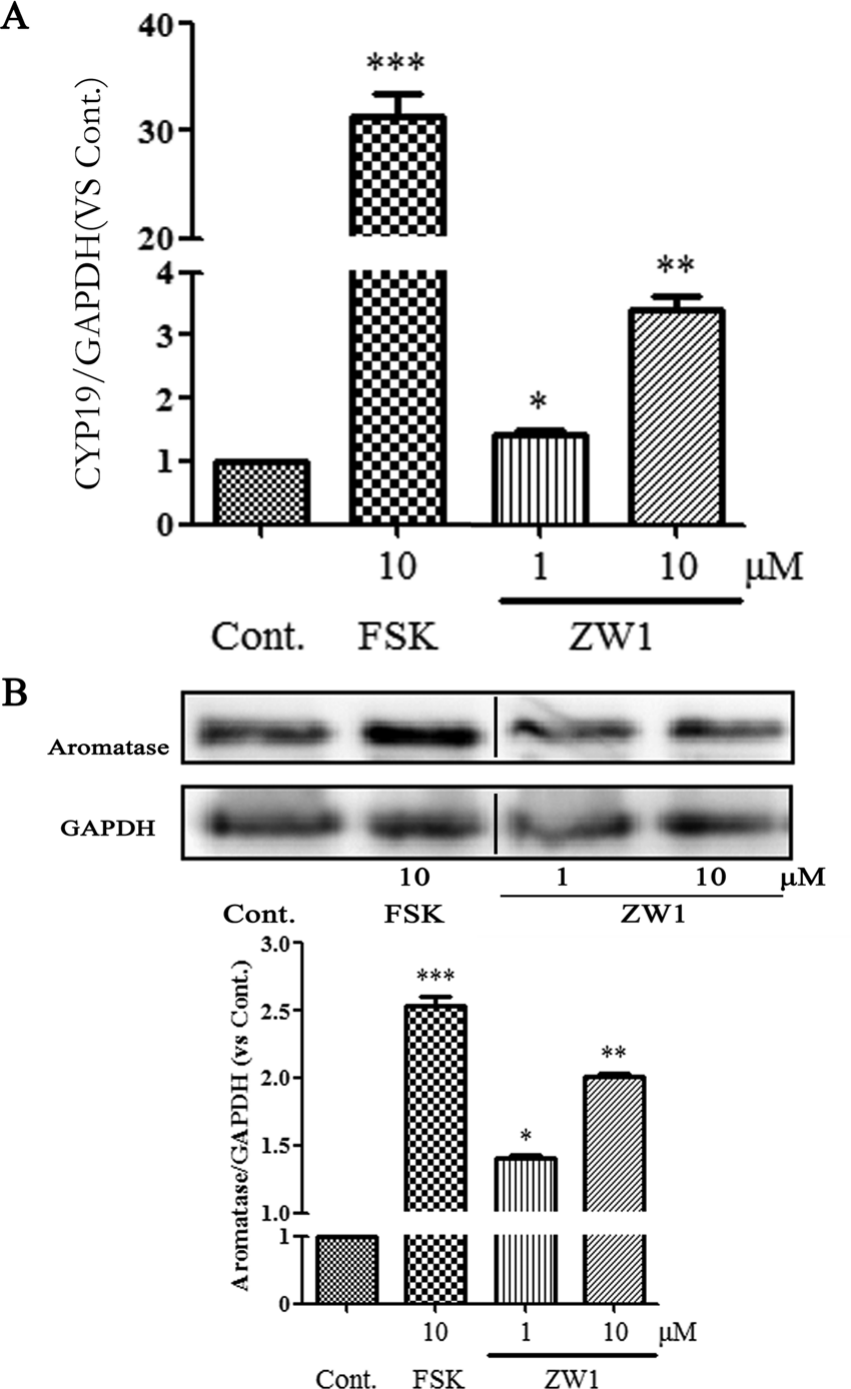
ZW1 promotes aromatase expression in KGN cells. KGN cells were treated with the test compounds at the indicated concentrations for 24 h. (**A**) Aromatase mRNA was measured in total cellular RNA by using real-time qPCR. The results were presented as the n-fold change relative to the basal levels in untreated cells. GADPH was used as an internal control. (**B**) The cell lysates were immunoblotted with an anti-aromatase or anti-GAPDH antibody. Cont., DMSO-treated control; FSK, 10 μM forskolin; LET, 1 μM letrozol. *p < 0.05, **p < 0.01, ***p < 0.001 compared with the control (*n* = 3). Black line represents one band removed, which was treated by formestane. The full western blot and the corresponding positions of the molecular weight protein markers are presented in Supplementary Fig. S2.

KGN cells lack endogenous 17α-hydroxylase and cannot synthesize androgens or estrogens de novo^35^. Therefore, the effect of ZW1 on 17β-estradiol production in KGN cells may be caused by aromatase, the only enzyme able to convert testosterone to 17β-estradiol. To investigate whether ZW1 enhancement of 17β-estradiol biosynthesis occurred through increased expression of aromatase, we examined the mRNA and protein levels of aromatase in KGN cells treated with ZW1. As shown in Fig. 5A, and consistent with previously reported results^36^, aromatase mRNA levels were significantly increased in KGN cells treated with forskolin. Aromatase transcription in KGN cells treated with 10 μM ZW1 increased approximately 3.5-fold when compared with that in control cells treated with DMSO. Comparable with their effects on aromatase transcription, ZW1 and forskolin increased aromatase protein expression. Compared with DMSO-treated control cells, addition of 10 μM ZW1 increased protein expression approximately 1.9-fold (Fig. 5B). These results indicated that ZW1 enhanced estrogen biosynthesis in KGN cells by increasing the expression of aromatase.

### ZW1 enhances CREB phosphorylation

**Figure 6:**
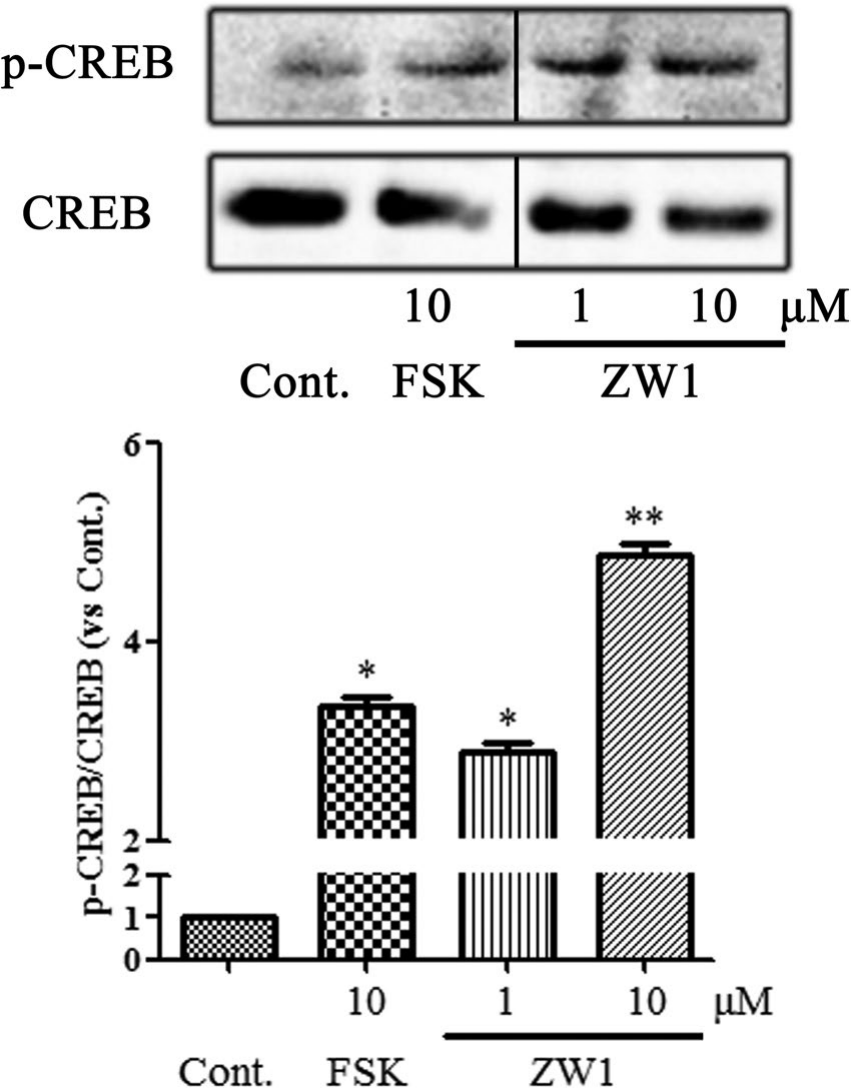
ZW1 promotes phosphorylation of CREB in KGN cells. KGN cells were treated with the test compounds at the indicated concentrations for 24 h. The cell lysates were immunoblotted with an anti-p-CREB or anti-CREB antibody. The relative amount of p-CREB to CREB protein was determined from the chemiluminescence intensities. The data are presented as mean ± SD. Cont., DMSO-treated control; FSK, 10 μM forskolin. *p < 0.05, **p < 0.01 compared with the control (*n* = 3). Black line represents one band removed, which was treated by letrozole. The full western blot and the corresponding positions of the molecular weight protein markers are presented in Supplementary Fig. S3.

CREB is a key transcription factor in ovarian granulosa cells recognizing the specific binding sites in promoter II to promote aromatase transcription in response to cAMP signaling^37, 38^. Therefore, we examined whether ZW1 could affect CREB. As shown in Fig. 6, and consistent with previous reports^39^, forskolin significantly enhanced CREB phosphorylation by over 3-fold. CREB phosphorylation was increased about 2.6- and 4.8-fold by addition of 1 and 10 μM ZW1, respectively. These results indicated that ZW1 increased the activation of CREB to promote aromatase expression.

### ZW1 promotes estrogen biosynthesis through PDE5 inhibition

**Figure 7:**
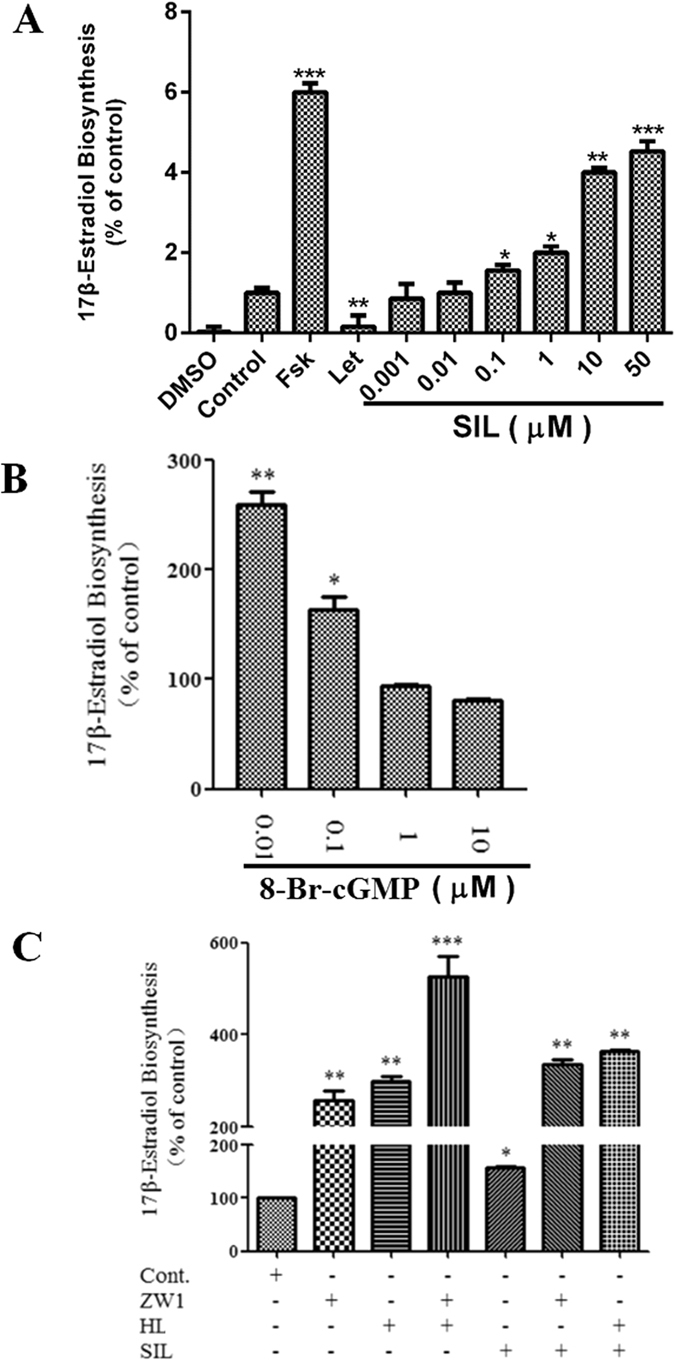
ZW1 promotes estrogen biosynthesis through inhibition of PDE5. (**A**) KGN cells seeded in 24-well plates and left overnight were pretreated with sildenafil at the indicated concentrations for 24 h, and then treated with 10 nM testosterone for a further 24 h. The concentration of 17β-estradiol in the culture medium was quantified using a 17β-estradiol magnetic particle-based ELISA. FSK, 10 μM forskolin; LET, 1 μM letrozol. (**B**) KGN cells were treated with 8-Br-cGMP at the indicated concentrations for 24 h, and then incubated with 10 nM testosterone for a further 24 h. The concentration of 17β-estradiol in the culture medium was quantified using a 17β-estradiol magnetic particle-based ELISA. (**C**) KGN cells seeded in 24-well plates and left overnight were treated with the test compounds for 24 h, and then 10 nM testosterone for a further 24 h. The concentration of 17β-estradiol in the culture medium was quantified using a 17β-estradiol magnetic particle-based ELISA. Cont., DMSO-treated control; ZW1, 10 μM ZW1; HL, 1 μM HL-725; SIL, 1 μM sildenafil. *p < 0.05, **p < 0.01, ***p < 0.001 compared with the control (*n* = 3).

It has been reported that sildenafil can enhance phosphorylation of CREB by activation of the cGMP pathway^40, 41^, suggesting PDE5 inhibition may promote estrogen biosynthesis through activation of the CREB-aromatase pathway. To demonstrate this, KGN cells were treated with various concentrations of sildenafil. The results shown in Fig. 7A indicate that sildenafil promoted 17β-estradiol biosynthesis in KGN cells in a concentration-dependent manner, suggesting the involvement of PDE5 in the regulation of estrogen biosynthesis. As inhibition of PDE5 increases intracellular cGMP levels, we further examined the effect of cGMP on estrogen biosynthesis in KGN cells. As shown in Fig. 7B, 0.01–0.1 μM cGMP significantly increased the biosynthesis of estrogen in KGN cells. We also examined whether PDE5 participated in ZW1-promoted estrogen biosynthesis. As shown in Fig. 7C, ZW1 and sildenafil significantly promoted 17β-estradiol biosynthesis. Treatment with a combination of ZW1 and sildenafil increased 17β-estradiol production 3-fold compared with sildenafil alone, suggesting that both compounds interact synergistically on 17β-estradiol production. PDE3 is a cAMP-hydrolyzing phosphodiesterase that is inhibited by cGMP^42^. To examine whether PDE3 participates in the cross-talk between cGMP and cAMP, we treated KGN cells with HL-725, a specific PDE3 inhibitor. Treatment with only HL-725 significantly increased 17β-estradiol production. Co-administration of ZW1 or sildenafil with HL-725 also significantly promoted the 17β-estradiol production with more potency than treatment with the individual compounds (Fig. 7C). These results suggested regulation of estrogen biosynthesis in KGN cells by PDE5 occurs partly through PDE3.

## Discussion

*E. brevicornum* Maxim has been used for the treatment of estrogen deficiency-related diseases in traditional Chinese herbal medicines for over 2000 years. More than 260 compounds have been identified from the *Epimedium* genus. The major constituents of these compounds are prenyl-flavonoids such as icariin, icaritin, and icariside II, which are also important chemotaxonomic markers^12^. Modern pharmacological studies and clinical practice have demonstrated that *E*. *brevicornum* Maxim and its active compounds possess a wide range of pharmacological actions, in particular, anti-osteoporosis, anti-aging, anti-atherosclerosis, and anti-depressant activity^43^. We previously found that the EtOH and *n*-butanol fractions of *E. brevicornum* Maxim extracts can promote estrogen synthesis in KGN cells^14^, but it was unclear whether compounds other than icariin contributed to this effect. In the present study, we isolated four new icariin analogues **1**–**4** from these two fractions, which also could enhance estrogen biosynthesis in KGN cells. These four new compounds share an isopentenyl group with other estrogen biosynthesis-promoting compounds including icariin (**5**), icariside II (**13**), and icaritin-3-O-α-L-rhamnoside (**17**), suggesting that icariin analogues containing an isoprenyl group could be an important new source of estrogen biosynthesis agonists. Icaritin-3-O-α-L-rhamnoside (**17**) exhibited much better activity than icariside II (**13)**, indicating the incorporation of a hydroxyl group at C-13 could significantly improve activity. It was noteworthy that the flavone luteolin (**16)**, in contrast to the other compounds that are flavonol glycosides, inhibited the biosynthesis of estrogen. This was consistent with our previous report^13^, which suggested that hydroxylation or glycosidation at C-3 was essential for the promotion of the biosynthesis of estrogen. Based on the bioactivities and chemical structures of compounds **5**–**14**, it was concluded that the number of sugar residues and the type of functional group at C-4′ did not significantly affect the biological activity. Icariin (**5**) and icariside II (**13**) showed estrogen-like protective effects on bone stimulated by estrogen receptor-dependent osteoblastic functions^44, 45^. In the present study, we found that icariin and icariside II significantly increased estrogen biosynthesis in KGN cells, suggesting that the promotion of estrogen biosynthesis may be a novel cause of the anti-osteoporotic activity of icariin and icariside II.

PDE5 plays a key role in cGMP signaling, but its role in estrogen biosynthesis in the ovary is rarely studied. In this study, we found that the icariin derivative ZW1, a validated potent PDE5 inhibitor, promoted estrogen biosynthesis in KGN cells by enhancing aromatase expression in a similar manner to icariin. Interestingly, PDE5 inhibitors, such as tadalafil and sildenafil, have been found to stimulate aromatase expression in human adipocytes^46^. Therefore, icariin and its derivatives may promote estrogen production in KGN cells through the inhibition of PDE5 activity. Treatment with PDE5 inhibitor alone, or in combination with testosterone, was found to improve sexual dysfunction in women^47–49^. The observations in this study provide a new insight into the explanation of the beneficial effect of PDE5 inhibitors in women who suffer sexual dysfunction by upregulation of aromatase expression in ovarian granulosa cells and stimulation of estrogen production. Previously, the beneficial effects of icariin and derivatives on female reproduction system development and sexual dysfunction were mainly attributed to their actions on estrogen receptors^50^. However, our data suggests that icariin and its derivatives may exhibit their beneficial effects by upregulation of estrogen biosynthesis through the inhibition of PDE5. Currently, the only clinically used agonist of estrogen biosynthesis is FSH. However, the use of protein-based FSH has some drawbacks, such as high cost, time-consuming production, and the stability and immunogenicity issues associated with a large protein drug. Compared with FSH, fractions rich with isoprenylated flavonoids or ZW1 can be easily obtained in large quantities from *E. brevicornum* or chemical synthesis, which makes them suitable for further investigation as therapeutic candidates for estrogen deficiency-related diseases.

The second messengers cAMP and cGMP are important regulators of estrogen production. In ovarian granulosa cells, cAMP-PKA-CREB is the classical pathway for transcriptional regulation of aromatase^51^. Other studies have also found that cGMP-cAMP crosstalk may also be involved in the regulation of ovarian function and expression of aromatase^6, 9^. Our study showed that ZW1 upregulated aromatase expression by activation of CREB phosphorylation; this was consistent with previous reports that PDE5 inhibitors could stimulate CREB phosphorylation in B16 melanoma cells *in vitro* and an Alzheimer’s disease mouse model *in vivo* ^41, 52^. In this study, we found that a low concentration of cGMP was able to increase the biogenesis of estrogen in KGN cells, supporting the observation that ZW1 and sildenafil promoted estrogen biogenesis in KGN cells. However, this was inconsistent with the suppression of estrogen accumulation by cGMP in rat ovarian granulosa cells, although it was also reported that cGMP-activated PKG could enhance aromatase expression and activity in cultured mice diencephalic neuron cells^53, 54^. This discrepancy may have been caused by the use of granulosa cells at different stages of differentiation or potential differences in species or culture conditions that may affect the cellular response to cGMP. In addition, cGMP signaling is fine-tuned spatially and temporally through the compartmentalization of PDEs^55^; therefore, different concentrations of cGMP may exhibit distinct roles in estrogen biosynthesis. cGMP may activate PKG to increase aromatase expression through CREB; however, it may also utilize classical cAMP signaling to regulate aromatase expression. PDE3 is a cGMP-inhibited cAMP-hydrolyzing PDE that can mediate the cross-talk between cGMP and cAMP signaling pathways^42^, but its role in the ovary is still unknown. In this study, we found that PDE3 inhibitors increased estrogen biosynthesis in KGN cells, suggesting it was involved in the estrogen production in granulosa cells. Therefore, an increased cGMP level resulting from PDE5 inhibition may suppress the cAMP-hydrolyzing activity of PDE3, which leads to an increased cAMP level in KGN cells; this activates the classical PKA-CREB pathway and promotes the expression of aromatase and the subsequent estrogen biosynthesis.

In summary, four new isoprenylated flavonoids and 16 known compounds were isolated and identified from the extract of *E. brevicornum*, and their effects on estrogen biosynthesis in human ovarian granulose-like KGN cells were evaluated. The isoprenylated flavonoid ZW1, a known PDE5 inhibitor, was used to examine the mechanism of these isoprenylated flavonoids on estrogen biosynthesis. ZW1 was found to significantly enhance estrogen biosynthesis in KGN cells in a concentration- and time-dependent manner. ZW1 upregulated the expression of aromatase through activation of CREB phosphorylation. Further investigation showed that the increased cGMP level resulting from ZW1-induced inhibition of PDE5 could promote estrogen biosynthesis in KGN cells, partly through the suppression of PDE3. These results not only help in understanding the clinical benefits and side-effects of *E. brevicornum* and constituent isoprenylated flavonoids, but also suggest that isoprenylated flavonoids may be an important new source of estrogen biosynthesis agonists. PDE5 warrants further investigation as a new therapeutic target for estrogen biosynthesis for the prevention and treatment of estrogen deficiency-related diseases.

## Methods

### General Experimental Procedures

Optical rotations were obtained on a Perkin-Elmer 341 polarimeter (Perkin-Elmer Corporation, Wellesley, MA, USA). High-resolution mass spectrometry was conducted on a LTQ Orbitrap XL mass spectrometer (Thermo Fisher Scientific, San Jose, CA, USA) with electrospray ionization sources operated in both positive and negative ion modes.^1^H and ^13^C NMR measurements were recorded on a Bruker Avance-600 spectrometer with tetramethylsilane (TMS) as the internal reference, and chemical shifts were expressed in δ (ppm). TLC was performed on pre-coated silica gel plates (GF254, 0.25 mm, Kang-Bi-Nuo Silysia Chemical Ltd, Yantai, China). Semi-preparative HPLC purifications were performed at 254 nm on a CXTH system equipped with a UV3000 detector (Beijing Chuangxintongheng Instruments Co. Ltd, Beijing, People’s Republic of China). Silica gel (200–300 mesh, Qingdao Marine Chemical Factory, People’s Republic of China) and Sephadex LH-20 (Pharmacia, Uppsala, Sweden) were used for column chromatography (CC).

### Plant Material

The aerial parts of *E. brevicornum* were purchased from Sichuan Neautus Traditional Chinese Medicine Co. Ltd, and identified by Prof. Weikai Bao of the Chengdu Institute of Biology, Chinese Academy of Sciences. A voucher specimen (YYH-2014-003) was deposited at the Laboratory of Phytochemistry, Chengdu Institute of Biology, Chinese Academy of Sciences.

### Extraction and Isolation

The aerial parts of *E. brevicornum* (30.0 kg) were powdered and extracted with 30% EtOH (100 L × 48 h × 3). The extract solution was evaporated under reduced pressure to remove EtOH and then diluted with distilled water. The diluted solution was passed through an HPD-100A macroporous resin column (300 mm × 1000 mm) and successively eluted with water and 80% EtOH. The 80% EtOH eluent was concentrated to yield a residue (2.4 kg), which was suspended in water and then successively extracted with EtOAc and *n*-BuOH. The *n*-BuOH extract (500.0 g) was fractionated by CC (silica gel 5.0 kg, EtOAc/CH_3_OH step gradients: 100:0, 90:10, 75:25, 60:40, and 50:50) to yield fractions 1–5. An aliquot of 0.8 g of fraction 2 (33.8 g) was purified by semi-preparative HPLC (CH_3_CN/H_2_O, 28:72) to yield compounds **5** (218.3 mg) and **10** (7.5 mg). The filtrate of fraction 3 (58.2 g) was subjected to Sephadex LH-20 CC (500 g, MeOH/H_2_O gradient from 20:80 to 60:40) and further purified by semi-preparative HPLC (CH_3_CN/H_2_O, 28:72) to yield compounds **1** (12.1 mg), **8** (33.0 mg), and **9** (19.2 mg). Fraction 4 (41.3 g) was further fractionated by silica gel CC (500 g, CHCl_3_/CH_3_OH step gradients: 10:1, 10:2, 10:3, 10:4, and 10:5) to yield six sub-fractions (fractions 4.1–4.6). Compounds **6** (8.8 mg), **7** (21.0 mg), **12** (38.5 mg), and **14** (15.1 mg) were isolated from fraction 4.4 (9.3 g) by semi-preparative HPLC (CH_3_CN/H_2_O, 27:73). Fraction 5 (33.9 g) was divided into five sub-fractions (fractions 5.1–5.5) by RP-18 CC (250 g, MeOH/H_2_O gradient from 30:70 to 70:30). Fraction 5.4 (2.1 g) was subjected to semi-preparative HPLC (CH_3_CN/H_2_O, 25:75) to afford compounds **11** (62.4 mg) and **20** (5.6 mg). The EtOAc extract (300 g) was subjected to CC on silica gel (3.0 kg) with a 20–80% gradient of EtOAc in petroleum ether, and ten fractions (fractions 1′−10′) were collected. A solid precipitate was obtained from fraction 3′ (3.8 g) and recrystallized from MeOH to give compound **16** (163.7 mg). Fraction 5′ (18.3 g) was purified by Sephadex LH-20 CC (300 g) with a 20–70% gradient of MeOH in H_2_O, and subsequently recrystallized in MeOH to yield compounds **3** (11.1 mg), **4** (16.3 mg), and **13** (48.9 mg). Fraction 6′ (25.3 g) yielded compounds **2** (12.9 mg), **15** (86.1 mg), **17** (10.3 mg), and **19** (135.9 mg) after Sephadex LH-20 CC (300 g) and semi-preparative HPLC (CH_3_CN/H_2_O, 28:72). Compound **18** (56.6 mg) was isolated from fraction 7′ (12.6 g) by silica gel CC (200 g, CHCl_3_/MeOH gradient from 10:1–10:3) and Sephadex LH-20 CC (MeOH/H_2_O gradient from 20:80 to 50:50).

### Structural Identification of the Sugar Residues

Compound **1** (6 mg) was dissolved in 2% H_2_SO_4_ solution (2 mL) and heated under reflux for 12 h. The reaction solution was extracted with EtOAc (5 mL × 3) to remove the aglycone. The H_2_O layer was neutralized with Ba(OH)_2_, filtered, and compared with authentic sugars by TLC analysis. Compounds 2–4 (each 3 mg) were mixed and processed as above. The absolute configuration of sugars was determined as follows: The filtered water solution of compound **1** was dried under reduced pressure to give a residue (3.0 mg), which was dissolved in pyridine (0.4 mL) containing L-cysteine methyl ester hydrochloride (3.5 mg) and heated at 60 °C for 1 h. Then, phenylisothiocyanate (3.5 μL) was added and the mixture was heated at 60 °C for another 1 h. The reaction mixture was directly analyzed by reversed-phase HPLC performed on a Lab Alliance series III pump equipped with a Lab Alliance Model 201 UV detector. The column used was a C_18_ column (SinoChrom ODS-BP, 4.6 mm × 250 mm, 5 μm, Elite, Dalian, China) and the mobile phase was CH_3_CN-H_2_O (25:75, v/v) containing 50 mM H_3_PO_4_. The analytical HPLC was carried out at a flow rate of 0.8 mL/min and the column temperature was set at 40 °C. From the hydrolysate of compound **1**, D-glucose and L-rhamnose were identified by comparison of the retention times of their derivatives with those of the authentic sugars derivatized in the same way, which showed retention times of 20.47 and 16.72 min, respectively. In compounds **2**–**4**, only L-rhamnose was detected.

### Materials

Icariin derivative ZW1 was synthesized as previously described^31^ and dissolved in DMSO at a concentration of 50 mM. Testosterone, letrozol, sildenafil, FSK, and protease inhibitor cocktail were purchased from Sigma Chemical Co. (St. Louis, MO, USA) and dissolved in DMSO at a concentration of 100 mM. All solutions were stored at −20 °C. Antibodies to aromatase (CYP19A1) and GAPDH were purchased from Epitomics (Burlingame, CA, USA) and Proteintech (Rosemont, IL, USA), respectively. Rabbit monoclonal antibodies to Phospho-CREB (Ser133) and CREB (48H2) were purchased from Cell Signaling Technology (Beverly, MA, USA).

### Cell Culture

Human ovarian granulosa-like KGN cells were maintained in Dulbecco’s modified Eagle medium/Ham’s F-12 nutrient mix (DMEM/F-12) medium (Invitrogen, Carlsbad, CA, USA) supplemented with 5% (v/v) fetal bovine serum (GIBCO, Invitrogen), penicillin (100 units/mL), and streptomycin (0.1 g/L) in cell culture plates at 37 °C in a humidified atmosphere containing 5% CO_2_.

### Cell-based Estrogen Biosynthesis Assay

This assay was performed as previously described^56^. KGN cells were seeded in 24-well plates and left overnight. On the following day, the medium was replaced with serum-free medium and the cells were pretreated with the test chemicals for 24 h. Testosterone (10 nM) was then added to each well, and cells were incubated for a further 24 h. The culture medium and cell lysate were collected and stored at −20 °C. The levels of 17β-estradiol in the culture medium were quantified by use of a magnetic particle-based 17β-estradiol enzyme-linked immunosorbent assay (ELISA) according to the manufacturer’s instructions (Bio-Ekon Biotechnology, Beijing, China). The intensities were measured at 550 nm with a Verioskan Flash Multimode Reader (Thermo Scientific, Waltham, MA) and normalized to the total cellular protein content. Protein determination was conducted with a bicinchoninic acid (BCA) protein assay kit (Bestbio, Shanghai, China).

### Western Blotting

Cells cultured in 6-well plates were lysed with RIPA Lysis Buffer (Beyotime, Haimen, China) supplemented with protease inhibitor cocktail (Sigma). Samples containing equal amounts of protein (40 μg) were mixed with 5x SDS loading buffer, boiled for 5 min, and subjected to SDS-PAGE on a 10% electrophoresis gel. After electrophoresis, proteins were blotted onto nitrocellulose membranes, blocked with 5% milk, incubated with the primary antibody at 4 °C overnight and a horseradish peroxidase-conjugated secondary antibody (Santa Cruz Biotechnology, Santa Cruz, CA, USA), and then developed using enhanced chemiluminescence solution (Amersham Bioscience, Piscataway, NJ, USA).

### Quantitative Real-time RT-PCR

Total cellular RNA was isolated using TRIzol reagent according to the manufacturer’s instructions (Invitrogen). Total RNA (2 μg) was reverse-transcribed using Promega Reverse Transcriptase (Promega) with oligo (dT) primers. Equal amounts of cDNA (2 μL) were subjected to real-time quantitative PCR with the Supermix (Bio-Rad) using a PikoReal detection system (Thermo Fisher Scientific, Waltham, MA). The primer pairs used in the assays for aromatase and GAPDH were as follows: aromatase, 5′-ACCCTTCTGCGTCGTGTC-3′ (sense) and 5′-TCTGTGGAAATCCTGCGTCTT-3′ (antisense); GAPDH, 5′-CCACCCATGGCAAATTCCATGGCA-3′ (sense) and 5′-GGTGGACCTGACCTGCCGTCTAGA-3′ (antisense). The thermal cycling conditions comprised an initial denaturation step at 95 °C for 3 min, followed by 40 cycles of 95 °C for 30 s, 53 °C for 20 s, and 72 °C for 30 s. The relative quantity of aromatase mRNA was calculated by the ΔΔ*C*_t_ method using GAPDH as a reference amplification from the same sample.

### Statistical Analysis

All statistical analyses were carried out using GraphPad Prism 5.0 software. The results were expressed as the mean ± standard deviation of individual values from three independent experiments. One-way ANOVAs followed by Duncan’s multiple range tests were used for the statistical comparisons. A *p-*value less than 0.05 was considered statistically significantly.

## Electronic supplementary material

Supplementary Information
