# Supplementary material for: Flavonoid glycosides isolated from Epimedium brevicornum and their estrogen biosynthesis-promoting effects
Source: Sci Rep. 2017 Aug 10;7:7760. doi: 10.1038/s41598-017-08203-7 (PMC5552768; doi:10.1038/s41598-017-08203-7)
Supplement: Supplementary file 1 — Supplementary Information [file 41598_2017_8203_MOESM1_ESM.pdf]

Supplementary Information

Flavonoid glycosides isolated from *Epimedium brevicornum* and their estrogen biosynthesis-promoting effects

Fu Li[1, †], Bao-Wen Du[1,†], Dan-Feng Lu[1,3,7,†], Wen-Xuan Wu[1,7], Kanjana Wongkrajang[4], Lun Wang[1], Wen-Chen Pu[1], Chang-Lu Liu[5], Han-Wei Liu[6], Ming-Kui Wang[1,2, *] & Fei Wang[1,*]

[1] Chengdu Institute of Biology, Chinese Academy of Sciences, Chengdu 610041, P. R. China.

[2] Key Laboratory of Mountain Ecological Restoration and Bioresource Utilization and Ecological Restoration Biodiversity Conservation Key Laboratory of Sichuan Province, Chengdu Institute of Biology, Chinese Academy of Sciences, Chengdu 610041, P. R. China.

[3] Key Laboratory of Animal Models and Human Disease Mechanisms of Chinese Academy of Sciences & Yunnan Province, Kunming Institute of Zoology, Chinese Academy of Sciences, , Kunming 650223, P. R. China

[4] Department of Chemistry, Faculty of Science and Technology, Pibulsongkram Rajabhat University, Phitsanulok 65000, Thailand.

[5] Key Laboratory of Exploitation and Study of Distinctive Plants in Education Department of Sichuan Province，Sichuan University of Arts and Science, Dazhou 635000, P. R. China.

[6] Ningbo Entry-Exit Inspection and Quarantine Bureau Technical Center, Ningbo 315012, P.R. China.

[7] University of Chinese Academy of Sciences, Beijing 100049, P. R. China

Content

1. ^{1}H and ^{13}C NMR assignments of compounds **1-4**

2. NMR and MS spectra of compounds **1-4**

3. Chemical structures of compound **5-20**

4. Full western blots of Figure 5 and Figure 6

1. ^{1}H and ^{13}C NMR assignments of compounds 1-4

Table S1. NMR data of compound **1** (δ in ppm, J in Hz)

NO.	δ_H[a]	δ_C[a]	NO.	δ_H[a]	δ_C[a]
1			1″	5.23 d (1.2)	102.3 d
2		158.3 s	2″	3.61 m	70.5 d
3		134.6 s	3″	3.38 m	70.8 d
4		178.6 s	4″	3.14 m	71.6 d
5		159.5 s	5″	3.98 m	70.2 d
6	6.57 s	98.5 d	6″	0.80 d (6.0)	18.3 q
7		160.8 s	1‴	4.95 d (6.0)	101.0 d
8		108.7 s	2‴	3.27 m	73.8 d
9		153.3 s	3‴	3.32 m	77.0 d
10		106.8 s	4‴		71.7 d
11	3.51, 3.59 m	21.9 t	5‴	3.41 m	77.6 d
12	5.13 t (4.8)	122.6 d	6‴	3.44, 3.69 m	61.1 t
13		131.7 s			
14	1.58 s	25.9 q			
15	1.67 s	17.9 q			
1′		120.8 s			
2′	7.32 d (1.6)	115.9 d			
3′		145.9 s			
4′		149.3 s			
5′	6.83 d (8.4)	115.9 d			
6′	7.27 dd (8.4, 1.6)	120.7 d			

[a] NMR data were recorded at 600 MHz in DMSO-d_6.

Table S2. NMR data of compound **2** (δ in ppm, J in Hz)

NO.	δ_H[a]	δ_C[a]	NO.	δ_H[a]	δ_C[a]
1			1′		122.9 s
2		157.0 s	2′	7.92 d (8.6)	131.0 d
3		134.5 s	3′	7.08 d (8.6)	114.4 d
4		178.4 s	4′		161.7 s
5		159.4 s	5′	7.08 d (8.6)	114.4 d
6	6.28 s	98.7 d	6′	7.92 d (8.6)	131.0 d
7		162.7 s	1″	5.32 d (1.2)	102.1 d
8		104.5 s	2″	3.41 m	70.8 d
9		154.9 s	3″	3.09 m	71.0 d
10		104.6 s	4″	2.96 m	71.5 d
11	2.82 m	29.5 t	5″	3.98 m	70.5 d
12	4.17 m	73.9 d	6″	0.71 d (4.4)	17.8 q
13		148.7 s	5-OH	12.61 s	
14	4.59 d, 4.68 d (3.6)	110.2 t	4′-OCH_3	3.83 s	55.9 q
15	1.65 s	17.8 q			

[a] NMR data were recorded at 600 MHz in DMSO-d_6.

Table S3. NMR data of compound **3** (δ in ppm, J in Hz)

NO.	δ_H[a]	δ_C[a]	NO.	δ_H[a]	δ_C[a]
1			1′		122.6 s
2		156.9 s	2′	7.87 d (6.0)	130.9 d
3		134.9 s	3′	7.10 d (6.0)	114.5 d
4		178.4 s	4′		161.8 s
5		162.1 s	5′	7.10 d (6.0)	114.5 d
6	6.29 s	93.9 d	6′	7.87 d (6.0)	130.9 d
7		166.8 s	1″	5.27 d (1.2)	102.4 d
8		104.5 s	2″	3.45 m	70.5 d
9		151.5 s	3″	3.17 m	71.1 d
10		105.0 s	4″	3.05 m	71.6 d
11	3.16 m, 3.26 m	26.4 t	5″	3.97 m	70.8 d
12	4.77 t (6.0)	92.1 d	6″	0.77 d (4.0)	17.9 q
13		70.5 s	5-OH	12.78	
14	1.13 s	26.1 q	4′-OCH_3	3.83	55.9 q
15	1.14 s	25.2 q			

[a] NMR data were recorded at 600 MHz in DMSO-d_6.

Table S4. NMR data of compound **4** (δ in ppm, J in Hz)

NO.	δ_H [a]	δ_C [a]	NO.	δ_H [a]	δ_C [a]
1			1′		122.3 s
2		157.4 s	2′	8.02 d (8.6)	131.2 d
3		135.8 s	3′	7.15 d (8.6)	114.6 d
4		179.3 s	4′		161.9 s
5		157.5 s	5′	7.15 d (8.6)	114.6 d
6	7.06 s	95.1 d	6′	8.02 d (8.6)	131.2 d
7		158.5 s	1″	5.37 d (1.2)	102.4 d
8		109.4 s	2″		70.5 d
9		148.7 s	3″		71.5 d
10		107.4 s	4″		71.2 d
11	7.00 s	97.6 d	5″	4.02 brs	70.7 d
12		164.7 s	6″	0.81 d (6.4)	17.9 q
13		67.8 s	5-OH	12.61 s	
14	1.54 s	29.3 q	4′-OCH_3	3.88 s	55.9 q
15	1.54 s	29.3 q			

[a] NMR data were recorded at 400 MHz in DMSO-d_6.

2. NMR and MS spectra of compounds **1**-**4**

Figure S1. ^{1}H-NMR spectrum of compound **1** in DMSO-*d6* (600 MHz)

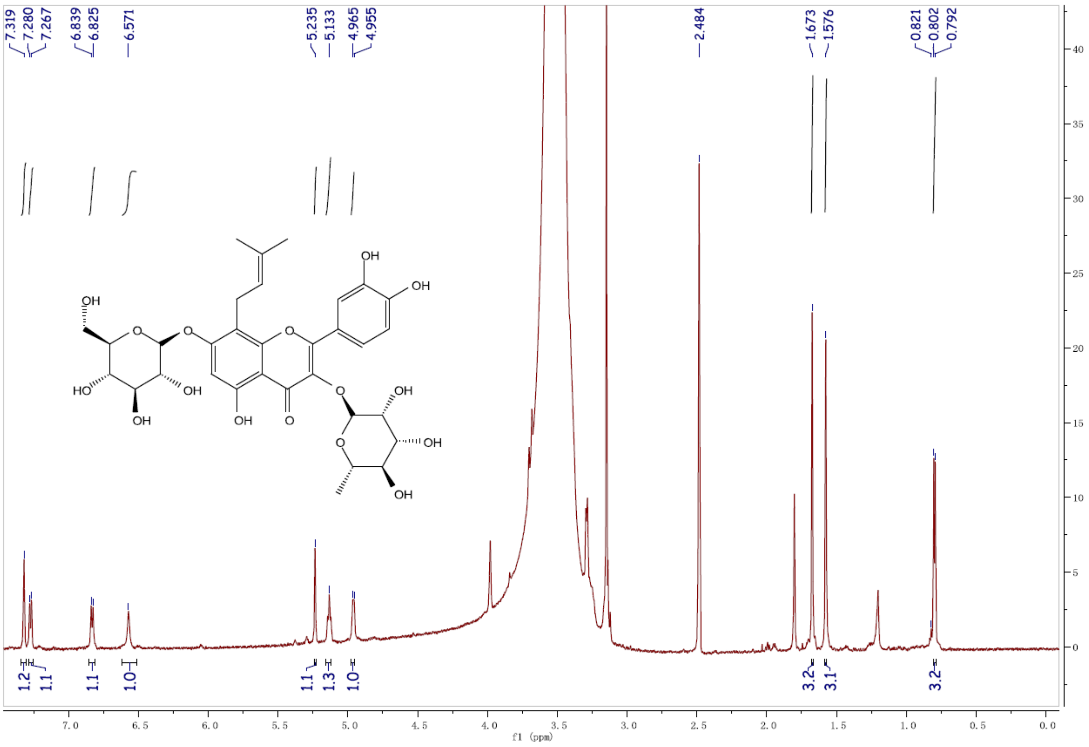

Figure S2. ^{13}C-NMR spectrum of compound **1** in DMSO-*d6* (150 MHz)

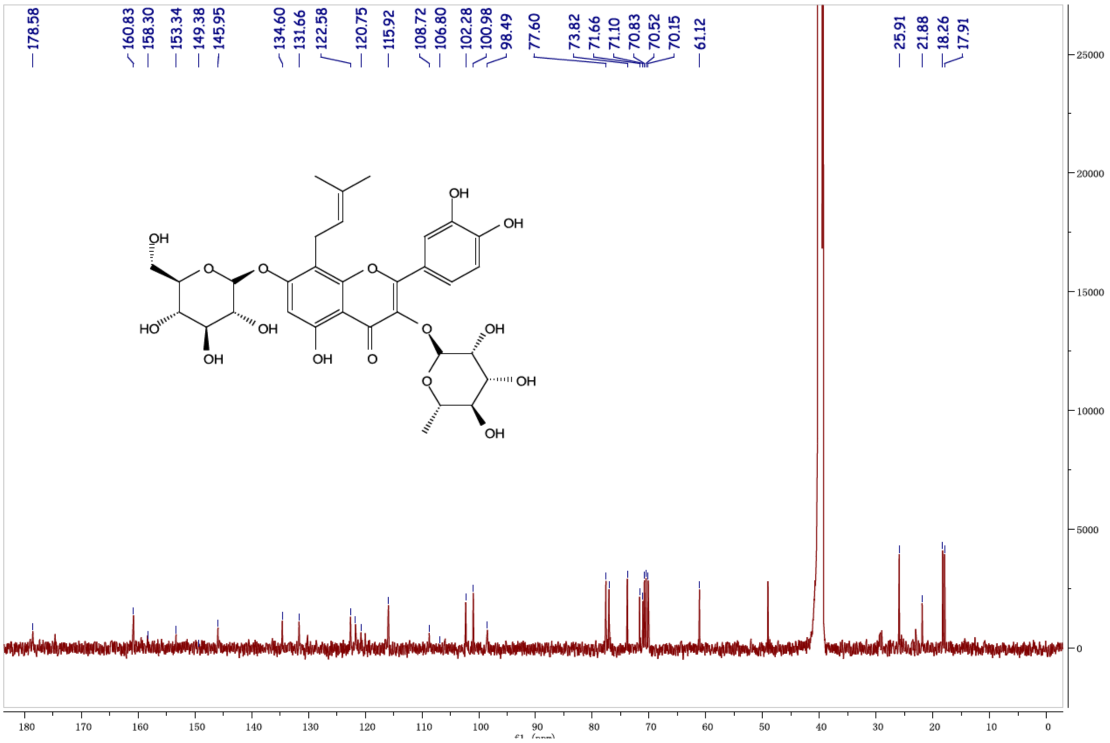

Figure S3. HSQC spectrum of compound **1**

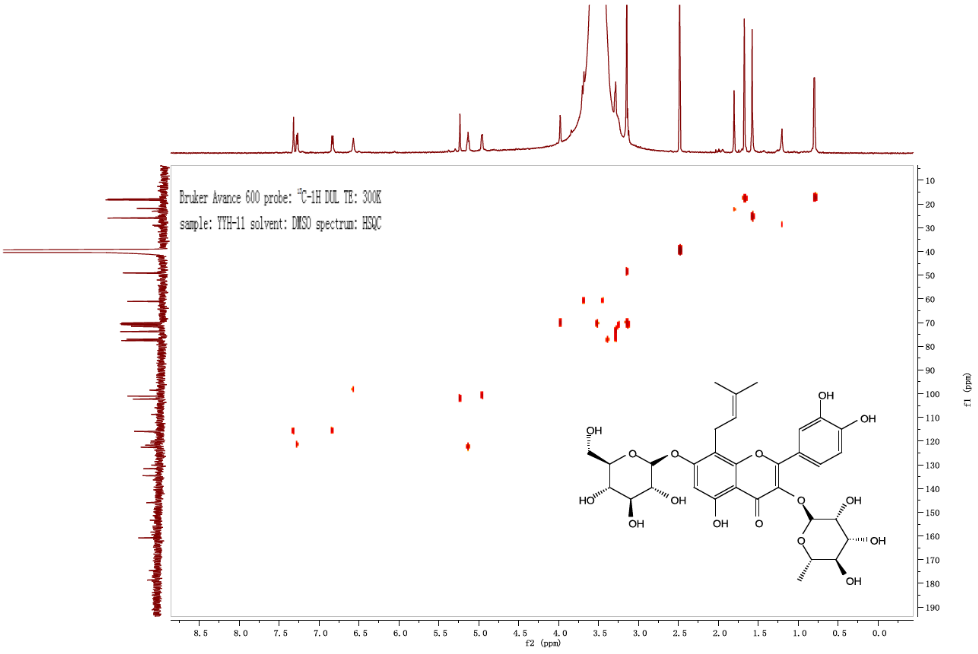

Figure S4. HMBC spectrum of compound **1**

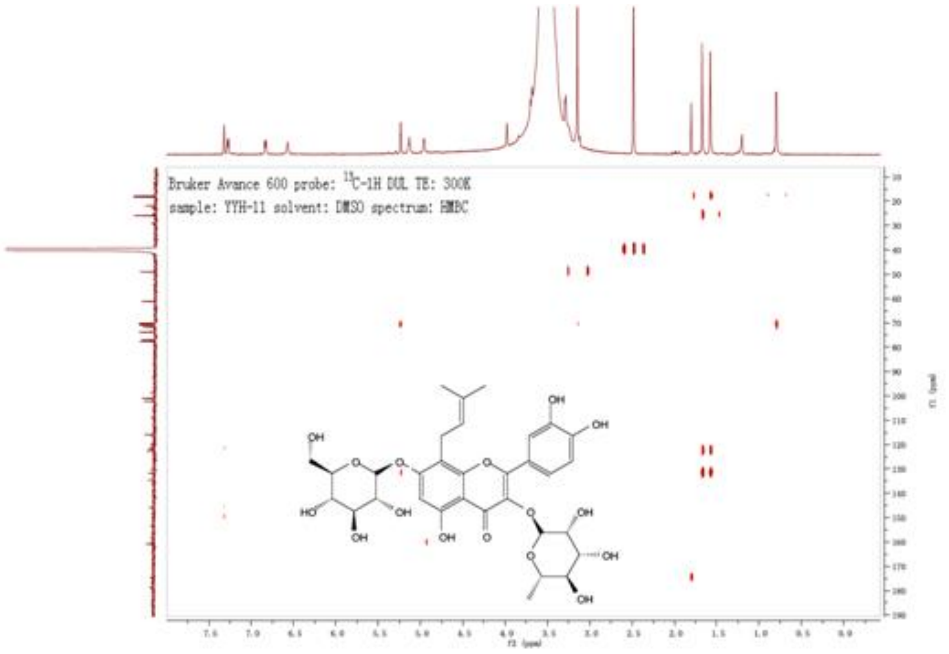

Figure S5. HR-ESI-MS spectrum of compound **1**

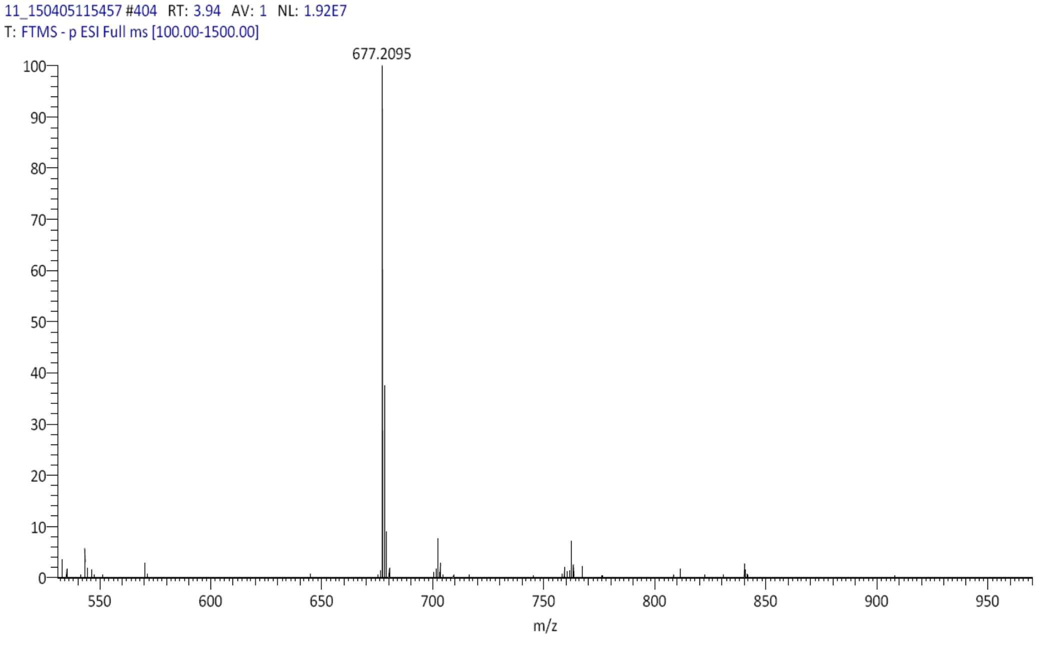

Figure S6. ^{1}H-NMR spectrum of compound **2** in DMSO-*d6* (600 MHz)

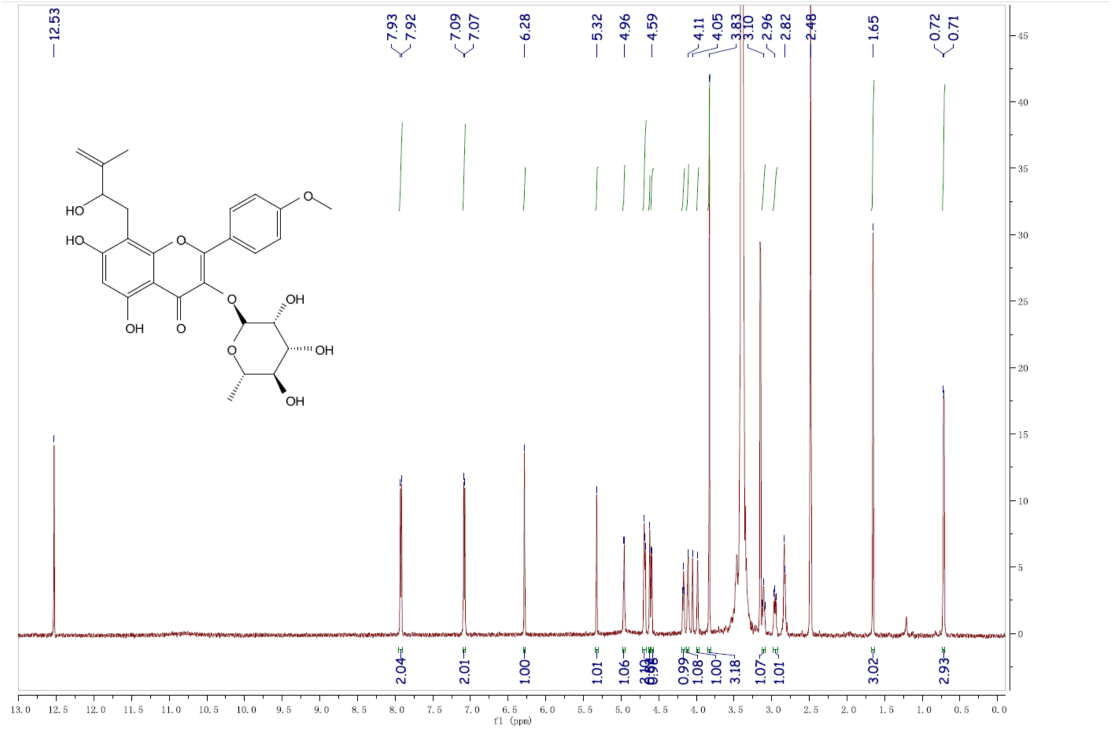

Figure S7. ^{13}C-NMR spectrum of compound **2** in DMSO-*d6* (150 MHz)

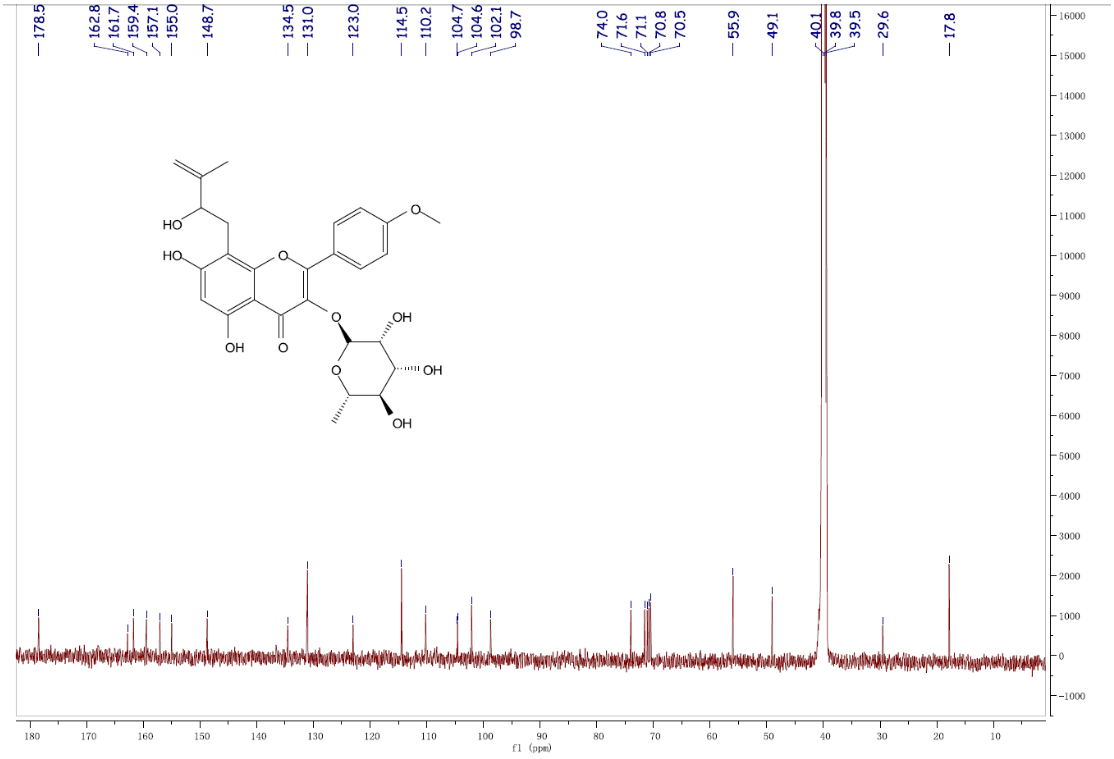

Figure S8. HSQC spectrum of compound **2**

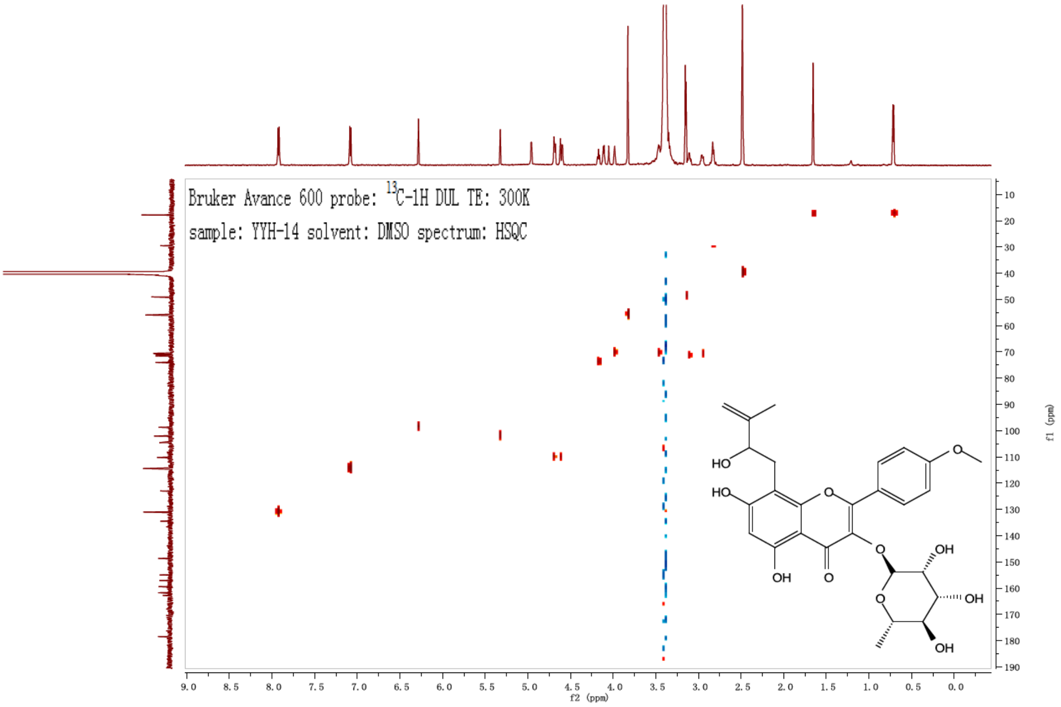

Figure S9. HMBC spectrum of compound **2**

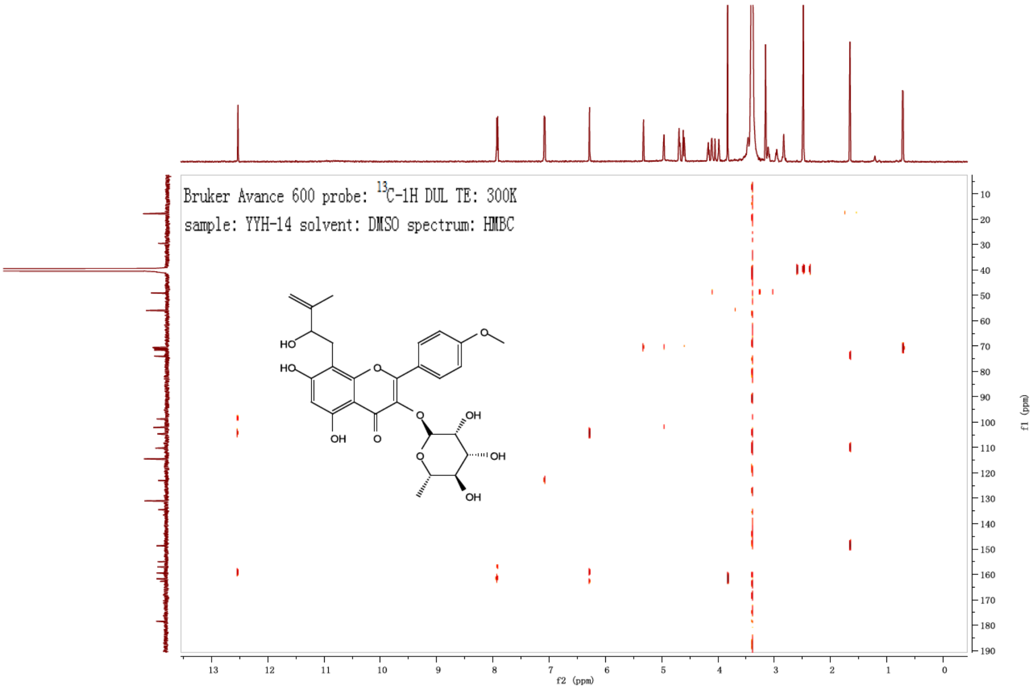

Figure S10. HR-ESI-MS spectrum of compound **2**

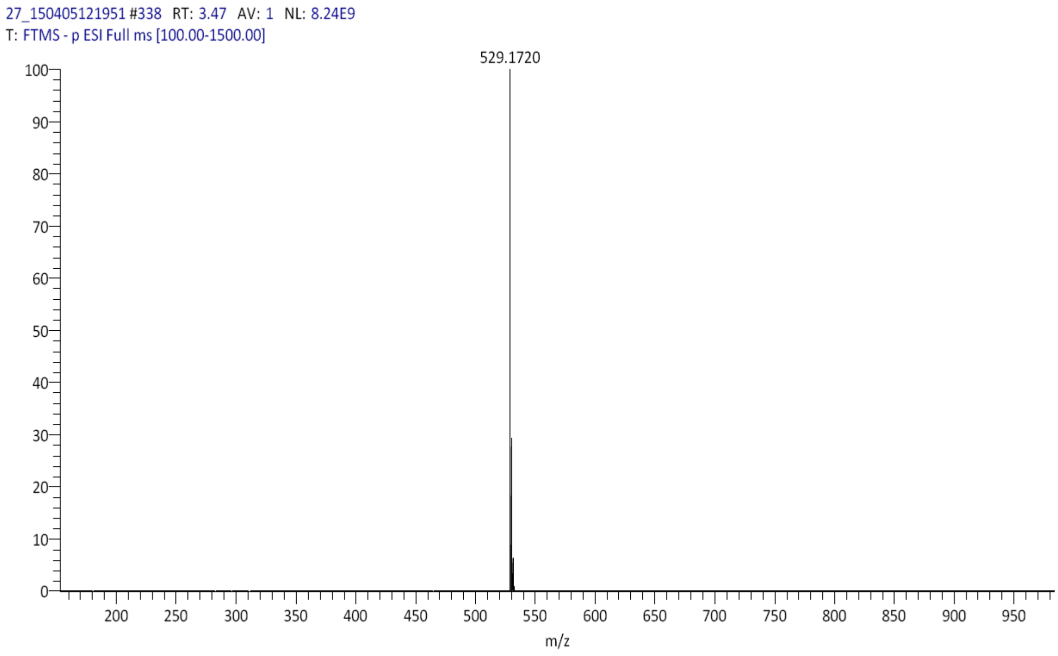

Figure S11. ^{1}H-NMR spectrum of compound **3** in DMSO-*d6* (600 MHz)

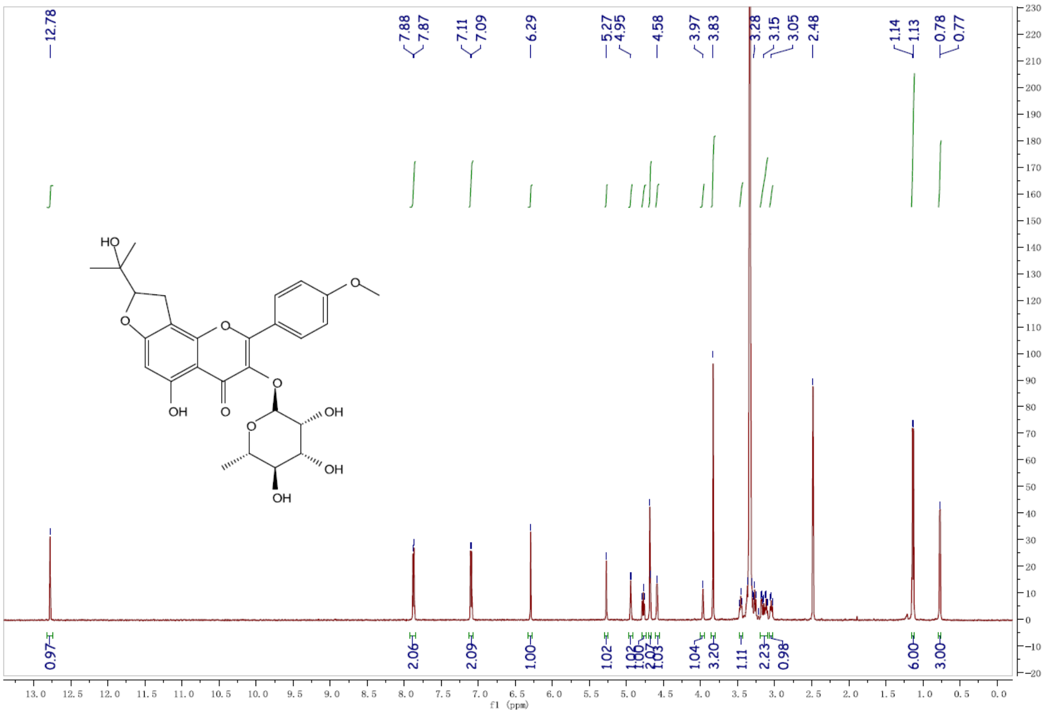

Figure S12. ^{13}C-NMR spectrum of compound **3** in DMSO-*d6* (150 MHz)

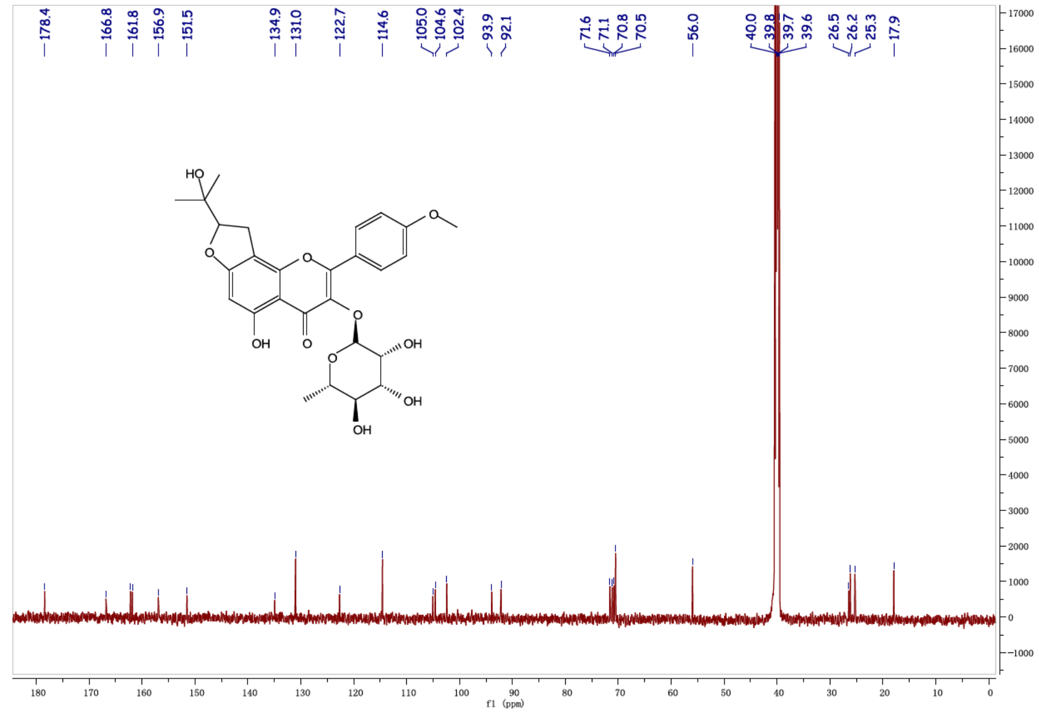

Figure S13. HSQC spectrum of compound **3**

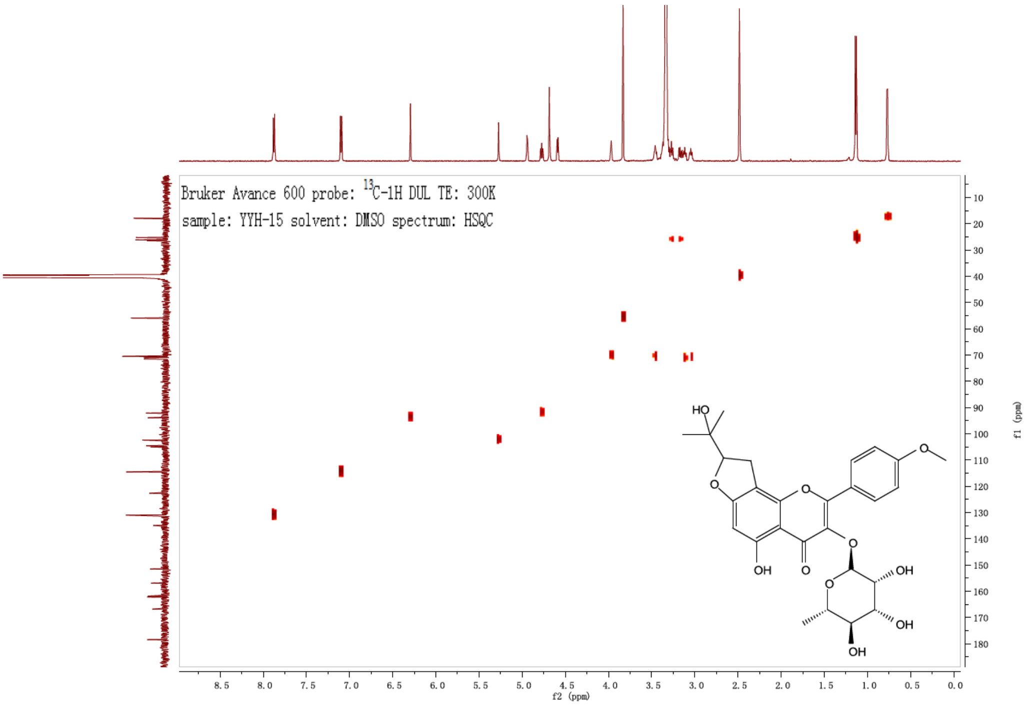

Figure S14. HMBC spectrum of compound **3**

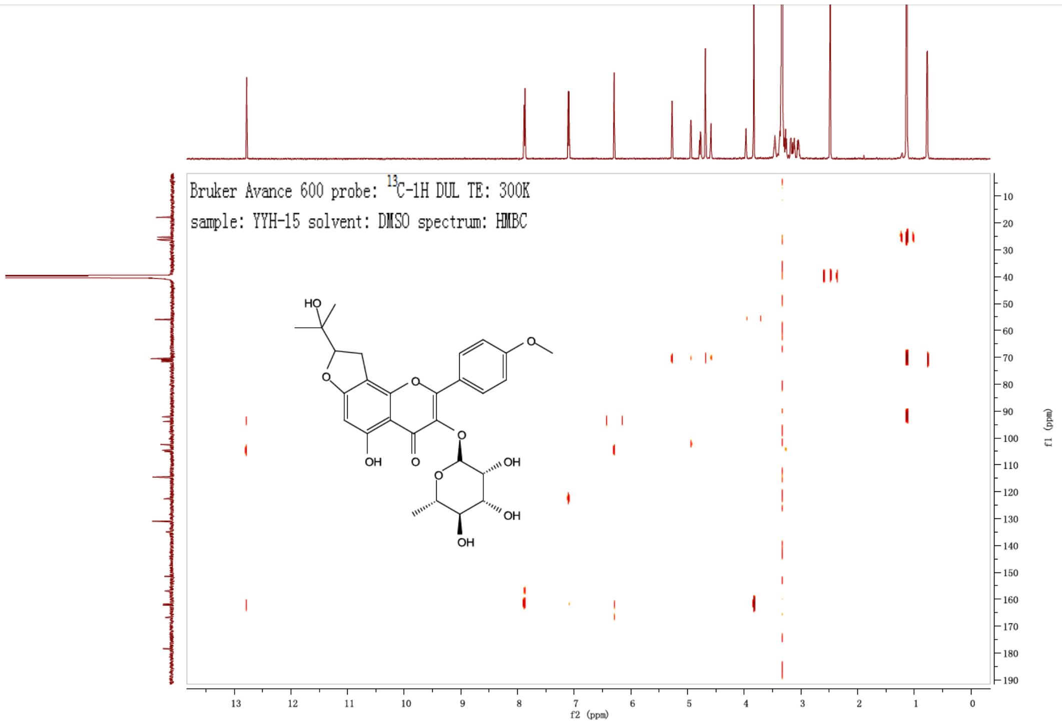

Figure S15. HR-ESI-MS spectrum of compound **3**

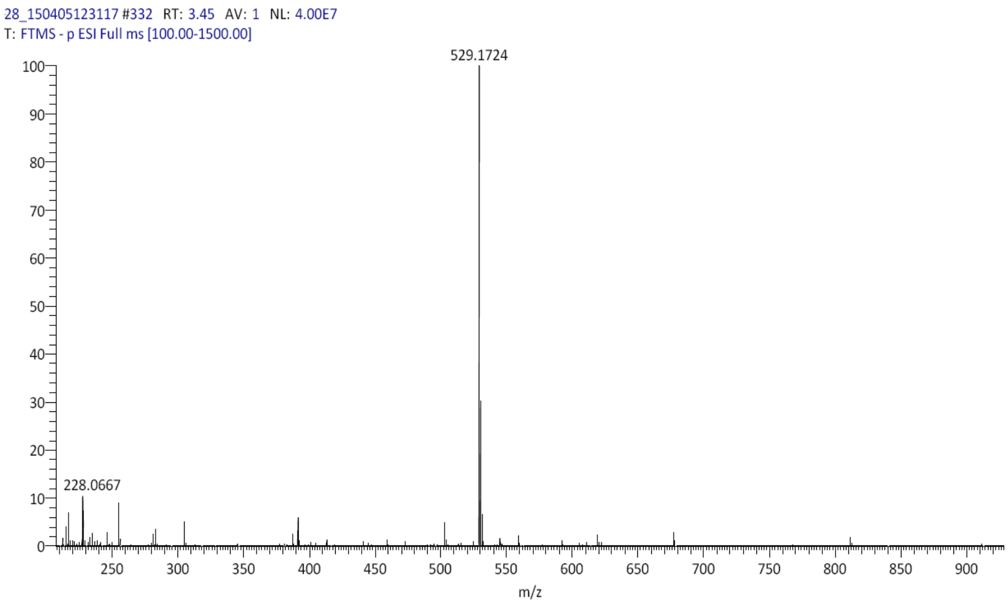

Figure S16. ^{1}H-NMR spectrum of compound **4** in DMSO-*d6* (400 MHz)

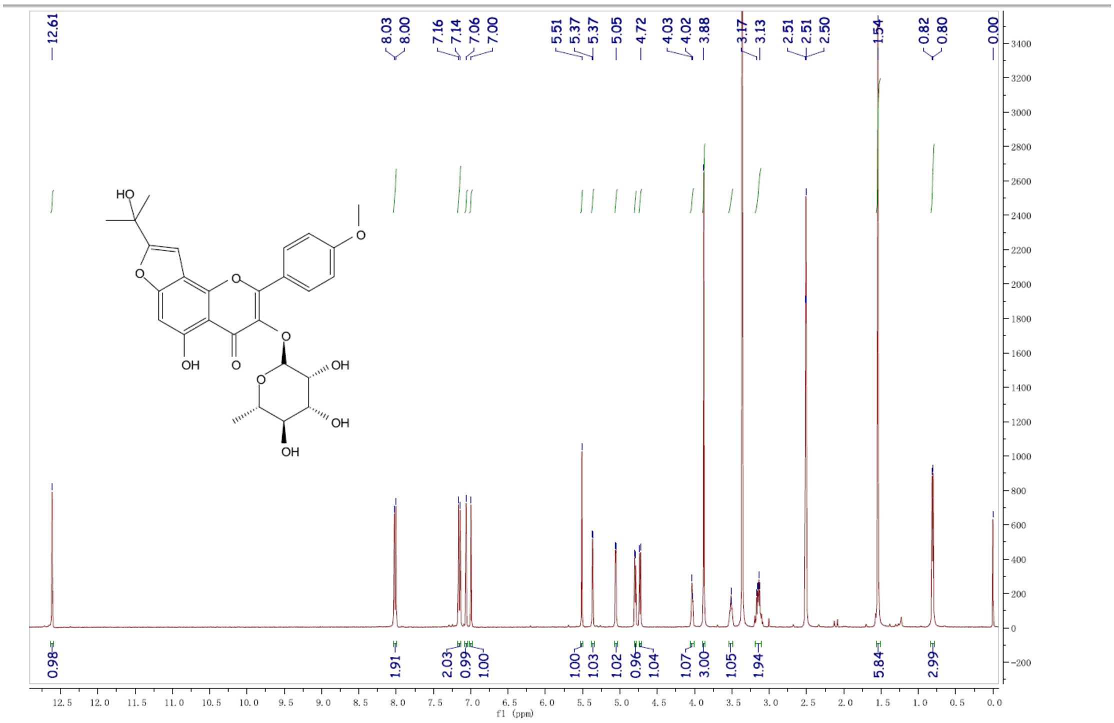

Figure S17. ^{13}C-NMR spectrum of compound **4** in DMSO-*d6* (100 MHz)

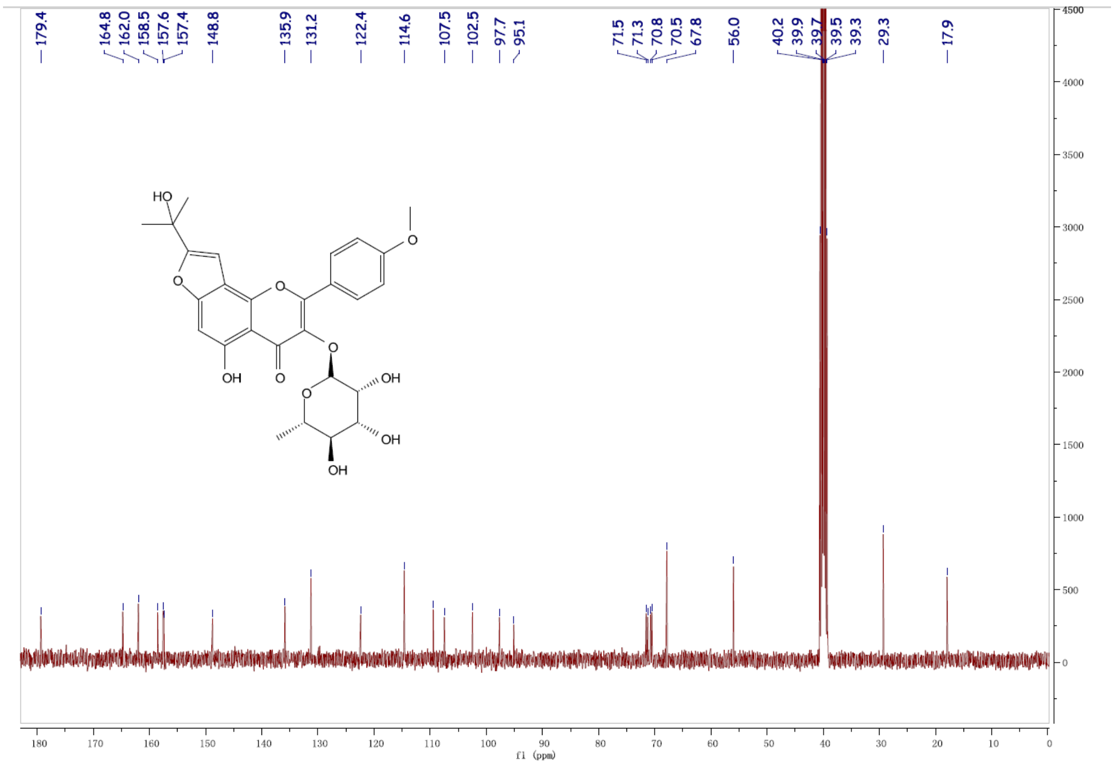

Figure S18. HSQC spectrum of compound **4**

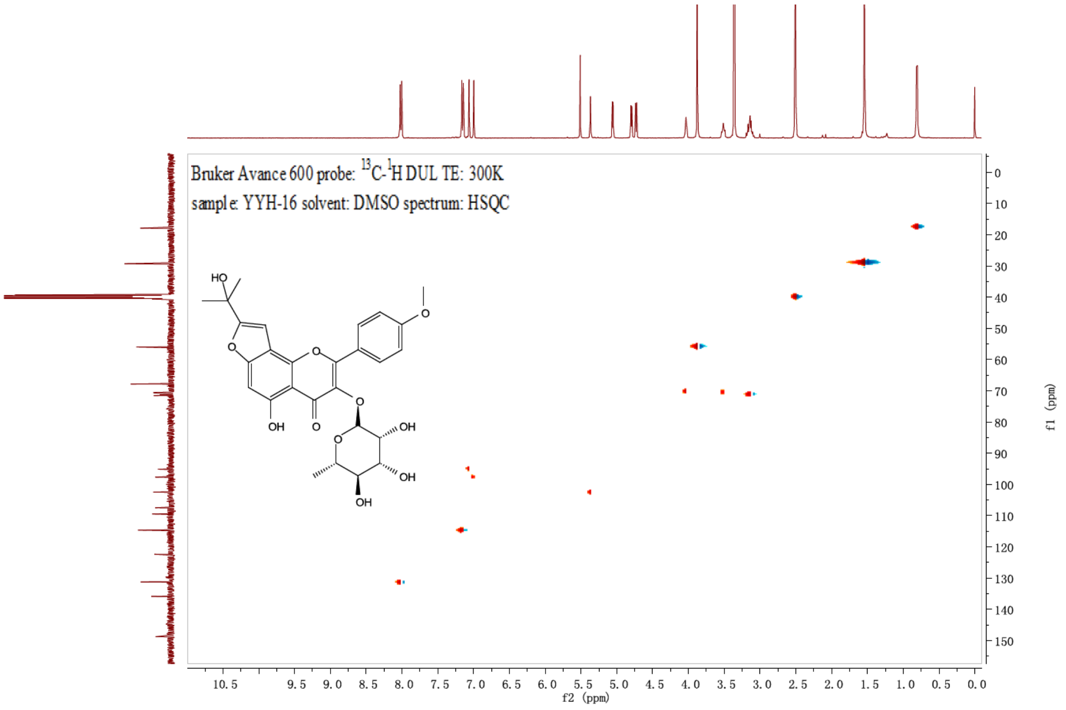

Figure S19. HMBC spectrum of compound **4**

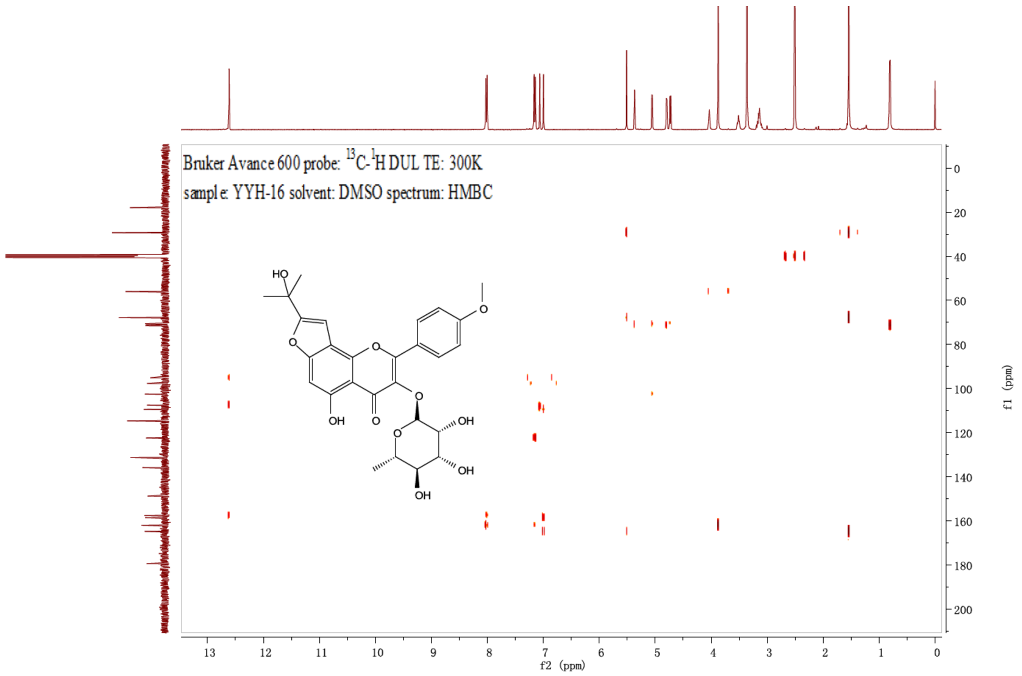

Figure S20. HR-ESI-MS spectrum of compound **4**

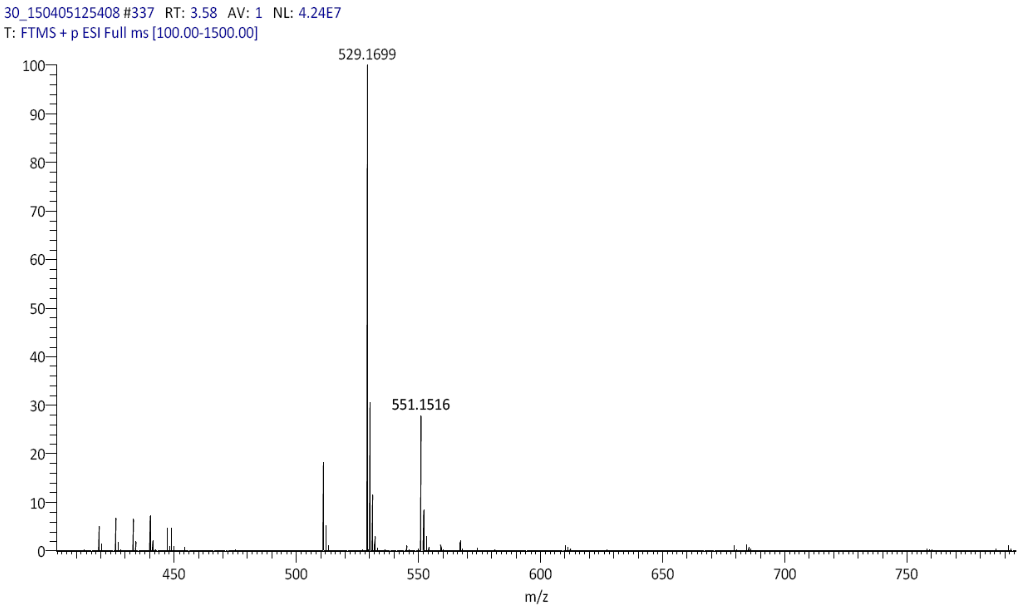

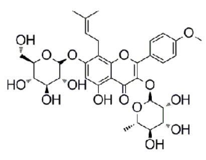
5

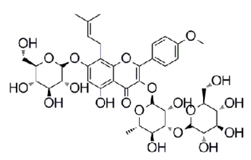
6

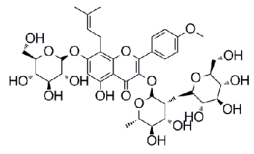
7

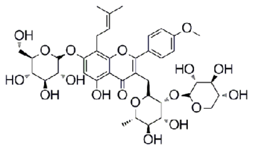
8

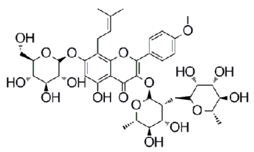
9

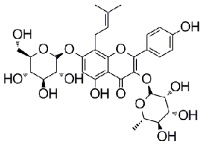
10

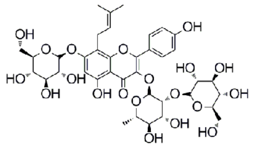
11

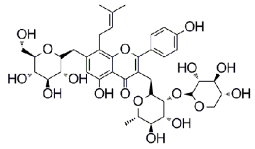
12

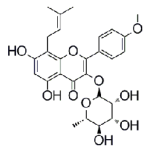
13

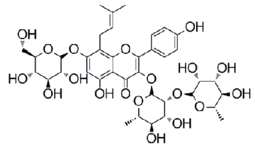
14

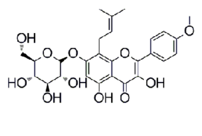
15

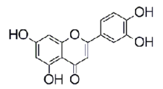
16

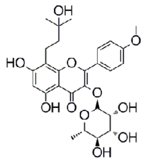
17

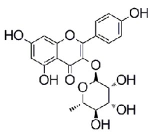
18

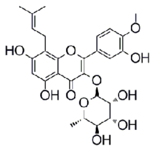
19

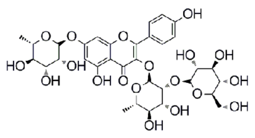
20

Figure S1 Chemical structures of compounds 5-20

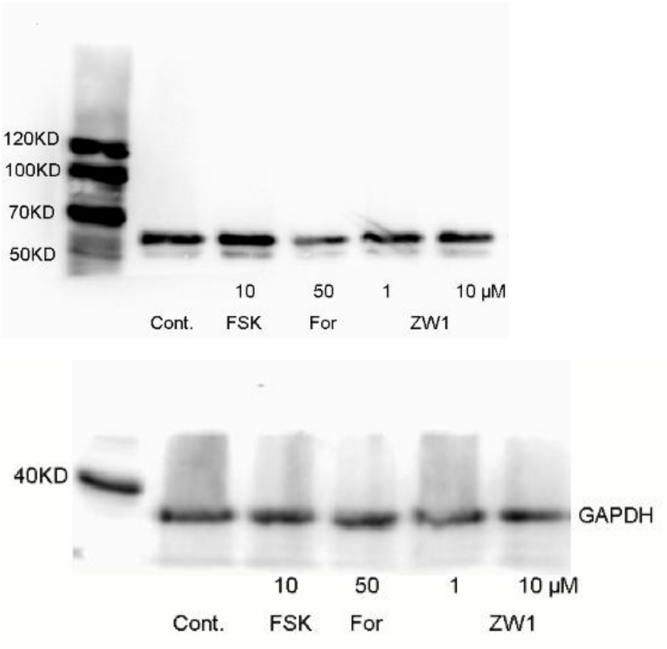

Figure S2 Full western blots of Figure 5B.

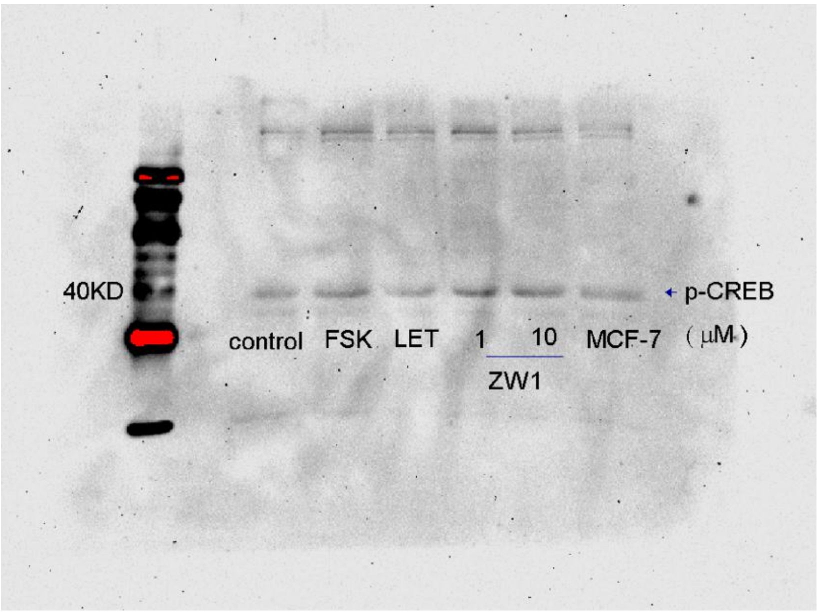

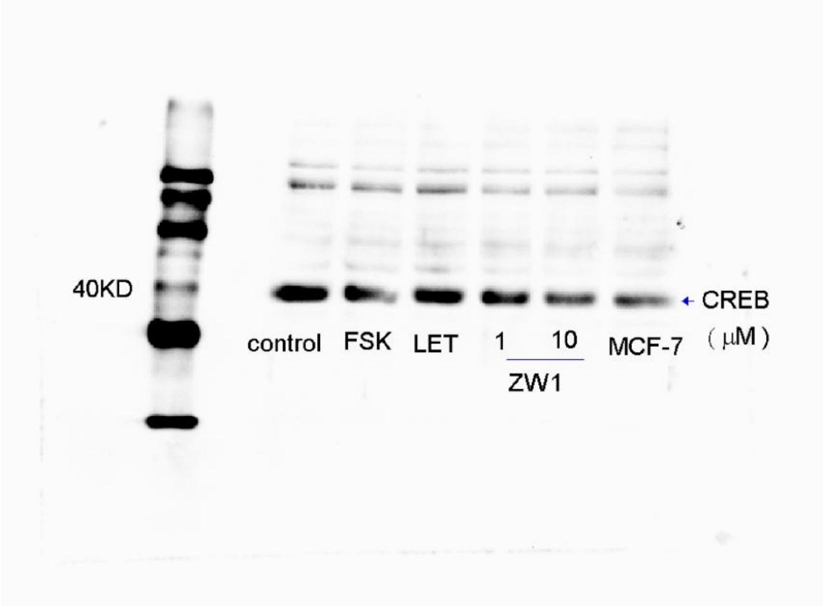

Figure S3 Full western blots of Figure 6.
